# Promoter Hypomethylation and Increased Expression of the Long Non-coding RNA LINC00152 Support Colorectal Carcinogenesis

**DOI:** 10.1007/s12253-020-00800-8

**Published:** 2020-04-20

**Authors:** Orsolya Galamb, Alexandra Kalmár, Anna Sebestyén, Titanilla Dankó, Csilla Kriston, István Fűri, Péter Hollósi, István Csabai, Barnabás Wichmann, Tibor Krenács, Barbara Kinga Barták, Zsófia Brigitta Nagy, Sára Zsigrai, Gábor Barna, Zsolt Tulassay, Péter Igaz, Béla Molnár

**Affiliations:** 1 2nd Department of Internal Medicine, Semmelweis University, Szentkirályi str 46, 1088 Budapest, Hungary; 2 MTA-SE Molecular Medicine Research Group, Hungarian Academy of Sciences and Semmelweis University, Budapest, Hungary; 3 1st Department of Pathology and Experimental Cancer Research, Semmelweis University, Budapest, Hungary; 4 Department of Physics of Complex Systems, Eötvös Loránd University, Budapest, Hungary

**Keywords:** LINC00152, CYTOR, Long non-coding RNA, Colorectal cancer, Colorectal adenoma

## Abstract

Up-regulation of the long non-coding RNA LINC00152 can contribute to cancer development, proliferation and invasion, including colorectal cancer, however, its mechanism of action in colorectal carcinogenesis and progression is only insufficiently understood. In this work we correlated LINC00152 expression with promoter DNA methylation changes in colorectal tissues along the normal-adenoma-carcinoma sequence and studied the effects of LINC00152 silencing on the cell cycle regulation and on the whole transcriptome in colon carcinoma cells using cell and molecular biology techniques. LINC00152 was significantly up-regulated in adenoma and colorectal cancer (p < 0.001) compared to normal samples, which was confirmed by real-time PCR and *in situ* hybridization. LINC00152 promoter hypomethylation detected in colorectal cancer (p < 0.01) was strongly correlated with increased LINC00152 expression (r=-0.90). Silencing of LINC00152 significantly suppressed cell growth, induced apoptosis and decreased cyclin D1 expression (p < 0.05). Whole transcriptome analysis of LINC00152-silenced cells revealed significant down-regulation of oncogenic and metastasis promoting genes (e.g. YES proto-oncogene 1, PORCN porcupine O-acyltransferase), and up-regulation of tumour suppressor genes (e.g. DKK1 dickkopf WNT signalling pathway inhibitor 1, PERP p53 apoptosis effector) (adjusted p < 0.05). Pathway analysis confirmed the LINC00152-related activation of oncogenic molecular pathways including those driven by PI3K/Akt, Ras, WNT, TP53, Notch and ErbB. Our results suggest that promoter hypomethylation related overexpression of LINC00152 can contribute to the pathogenesis of colorectal cancer by facilitating cell progression through the up-regulation of several oncogenic and metastasis promoting pathway elements.

**Electronic supplementary material:**

The online version of this article (10.1007/s12253-020-00800-8) contains supplementary material, which is available to authorized users.

## Introduction

Colorectal cancer (CRC) is one of the leading death-causing malignant diseases worldwide with high mortality [1]. Malignant transformation of colorectal epithelia can develop through different molecular pathways accumulating genetic and epigenetic alterations [2]. Besides the conventional mutations, recent research activities have been focused on the epigenetic regulatory mechanisms such as DNA methylation, histone modification and noncoding RNAs in colorectal carcinogenesis and progression. Revealing the mechanism of some of these defects can help in understanding the molecular complexity of CRC development and may result in potential biomarkers of CRC behavior.

The number of studies testing the regulatory role of the non-translated genomic regions is exponentially increasing. Long non-coding RNAs (lncRNAs) belong to the largest class of non-protein-coding RNAs that are RNA polymerase II transcripts of > 200 nucleotides lacking an open reading frame [3]. LncRNAs can regulate gene expression, alternative splicing, protein activity and localization in various ways by interacting with DNA, RNA, and proteins [4–6].

LncRNAs contribute to the development of different malignomas including gastrointestinal cancers. Increased expression of LINC00152 (alias CYTOR) lncRNA was previously described in different gastrointestinal cancer tissues such as gastric cancer (GC) and hepatocellular carcinoma (HCC) compared to adjacent normal and healthy normal tissues [7–11]. Li *et al*. detected elevated plasma levels of LINC00152 both in plasma circulating nucleic acids and in exosomes of patients with GC [12]. LINC00152 is also considered as a potential plasma biomarker of HCC [13]. Neumann *et al*. found LINC00152 to be hypomethylated in HCC [14].

It has been reported that LINC00152 has an oncogenic function in GC cell lines by facilitating cell proliferation, epithelial-mesenchymal transition, promoting cell migration and invasion, while it inhibits cell cycle arrest at G1 phase and apoptosis [11]. LINC00152 can enhance cancer cell proliferation and tumour growth *in vivo*, as well [8, 9, 15]. LINC00152 exerts its cell proliferation promoting activity through different molecular pathways as observed in gastric, colonic and hepatocellular carcinoma cell lines [8, 9, 15–17]. It can directly bind to EGFR causing PI3K/Akt signalling activation [15] or can regulate cell cycle by recruiting enhancer of zeste 2 polycomb repressive complex 2 subunit to the p15 and p21 promoters, resulting in the silencing of these genes [8]. In HCC, LINC00152 contributes to the increasement of cell proliferation by activating mTOR signal transduction pathway through the elevation of EpCAM level [13]. LncRNAs can act as competing endogenous RNAs and play an important role in the regulation of cell proliferation-related genes via modulating miRNA levels [6]. Recently it has been published that LINC00152 stimulates the proliferation of CRC cells via miR193-3p/ERBB4/AKT [17] and miR139-5p/NOTCH1 [16] signalling axes and contributes to the induction/increase of chemoresistance, as well [16, 17].

Although the up-regulation of LINC00152 has already been reported in CRC compared to normal adjacent tissue (NAT) [16–18]; its expression, promoter DNA methylation and association with colorectal normal-adenoma-carcinoma sequence progression have not been clarified.

In this study, we aimed to analyse the expression, *in situ* localization of LINC00152 and DNA methylation changes of its promoter region during the colorectal normal-adenoma-carcinoma sequence progression. The effects on cell growth and the possible gene expression regulatory role of this lncRNA were also studied at whole transcriptome level in SW480 colon carcinoma cell line.

## Material and Methods

### Clinical Samples

Affymetrix HGU133Plus2.0 microarray data of 147 colonic biopsies (GSE4183 [19], GSE10714 [20] and GSE37364 [21]) and Human Transcriptome Array 2.0 data of 60 colonic biopsy specimens (GSE100179 [22]) were previously analysed. In this study, real-time PCR validation was performed on 30 of 147 biopsy samples (10 normals, 10 adenomas and 10 CRCs) and on 60 independent biopsies (20 normal, 20 adenomas and 20 CRCs) (Table 1). LINC00152 *in situ* hybridization was done on tissue microarray (TMA) slides including 5 normal, 29 adenoma and 20 CRC tissue samples (Table 1).

**Table 1:** Clinical samples involved in the study

	GENE EXPRESSION ANALYSIS			DNA METHYLATION ANALYSIS			
	Real-time PCR		*In situ* hybridization		*In silico* DNA methylation analysis		
	Original set	Independent set		Bisulfite sequencing	Methyl capture sequencing [23]	Illumina BeadChip450K GSE48684 [24]	Illumina BeadChip450K TCGA [25]
NAT/N	10	20	5 (5)	13	6	41	38 (38)
mean age ± SD (years)	53 ± 17	54 ± 16	54 ± 21	48 ± 16	69 ± 6	NA	69 ± 12
gender (F/M)	6/4	16/4	4/1	10/3	2/4	NA	17/21
AD	10	20	12 (29)	22	15	42	**-**
mean age ± SD (years)	68 ± 11	67 ± 10	68 ± 8	68 ± 10	67 ± 10	NA	
gender (F/M)	4/6	7/13	8/4	11/11	2/13	29/13	
histological type							
tubular	7	9	7	15	NA	NA	
tubulovillous	3	11	5	7	NA	NA	
dysplasia							
low-grade	9	18	12	19	9	NA	
high-grade	1	2	-	3	6	NA	
CRC	10	20	14 (20)	12	9	64	283 (301)
mean age ± SD (years)	71 ± 8	68 ± 10	72 ± 11	71 ± 9	70 ± 8	NA	65 ± 13
gender (F/M)	9/1	9/11	8/6	5/7	2/7	41/23	129/154
stage (AJCC 7^th^ edition)							
Stage I	2	3	2	3	2	Stage I or II:	43
Stage II	3	6	3	2	3	21	110
Stage III	2	5	4	4	2	Stage III or IV:	85
Stage IV	2	5	3	2	2	43	37
Unknown stage	1	1	2	1	-	-	8
localization							
Proximal colon	4	5	3	3	2	28	153
Transverse colon	-	1	3	-	-	3	27
Distal colon	-	7	4	7	4	25	99
Rectum	6	7	4	2	3	6	-
Unknown	-	-	-	-	-	2	4
Total number of samples	30	60	31 (54)	47	30	147	283 (339)

Our study was conducted according to the Helsinki declaration and approved by the local ethics committee and government authorities (Regional and Institutional Committee of Science and Research Ethics (TUKEB) Nr.: 69/2008, 202/2009, 23970/2011 Semmelweis University, Budapest, Hungary). All routine colonic tissue samples from the patients were taken after informed consent and ethical permission was obtained for participation in the study. In case of *in situ* hybridization and Illumina BeadChip450K TCGA studies, numbers of samples are represented in brackets after patient numbers, in other analyses the samples numbers were equal with patients numbers. NAT = normal adjacent tissue; N = healthy normal; AD = adenoma; CRC = colorectal cancer; NA = not available

Methyl capture sequencing (MethylCap-seq) data of 30 colonic samples (15 adenomas, 9 CRCs and 6 normal adjacent tissue (NAT)) [23] were evaluated (Table 1). Methylation status of LINC00152 promoter was analysed on 47 independent biopsies (13 healthy normals, 22 adenomas and 12 CRCs).

The study was conducted according to the Helsinki declaration and approved by the local ethics committee and government authorities (Regional and Institutional Committee of Science and Research Ethics (TUKEB) Nr.: 69/2008, 202/2009, 23970/2011 Semmelweis University, Budapest, Hungary). All routine colonic tissue samples from the patients were taken after informed consent and ethical permission was obtained for participation in the study. After informed consent of untreated patients, colonic biopsies were taken during endoscopic intervention and stored in RNALater Reagent (Ambion-Life Technologies, Carlsbad, California, USA) at − 80 °C until use. Biopsy samples from the same site were immediately fixed in buffered formalin for histological evaluation by experienced pathologists. Detailed patient specification is described in Supplementary Table S1.

### Quantitative Real-time PCR Analysis

Real-time PCR validation was performed on 90 fresh frozen, RNAlater stabilized biopsy samples (Table 1) as previously described [22]. The applied primer sequences were the following: LINC00152-forward: 5′-CTCCAGCACCTCTACCTGTTG-3′; LINC00152-reverse: 5′-GGACAAGGGATTAAGACACACA-3′ [12]. For statistical evaluation, Kruskal-Wallis variance test was applied to determine significance between the three analysed sample groups. For pairwise comparison, Mann-Whitney test with FDR correction was used and adjusted p-value of < 0.05 was considered as significant.

### *In situ* Hybridization and Immunofluorescence

*In situ* hybridization (ISH) experiments were performed on 5 µm thick 10% formalin-fixed, paraffin-embedded (FFPE) tissue sections and on TMA slides containing 5 normal colonic mucosa, 29 adenoma (from 12 adenoma patients) and 20 CRC (from 14 CRC patients) FFPE tissue sample cores (Table 1, Supplementary Table S1) in collaboration with Boye Schnack Nielsen, (Bioneer A/S, Hørsholm, Denmark) using the RNAscope ISH system in a Ventana Discovery Ultra instrument (Roche, Basel, Switzerland) [26]. The following RNAscope probes were incubated on tissue sections according to the manufacturers’ instructions: CYTOR (cytoskeleton regulator RNA (LINC00152), NR_024204.2, target region: 186–794, 11 zz pairs), dapB negative control probe (a Bacillus subtilis gene, 414–862, 10 zz pairs), and PPIB positive control probe (Cyclophilin B, 139–989, 16 zz pairs), all from ACD, Biotechne Co. (Newark, CA). These probes were detected using the horseradish-peroxidase (HRP) kit and Discovery-rhodamine substrate (Roche). For cytokeratin immunofluorescence, the AE1/3 mouse monoclonal antibody (1:200, Dako-Agilent, Glostrup, Denmark) was detected with the Alexa-488 conjugated anti-mouse Ig (Jackson Immunoresearch, West Grove, PA). The stained sections were mounted with a DAPI-containing anti-fade solution, ProLong Gold (ThermoFisher). The results were examined on digital whole slides obtained with the Pannoramic Confocal (3DHISTECH Ltd., Budapest, Hungary) digital slide scanner using a 40x objective (NA = 0.93). The evaluation of the TMA slides has been done by three independent experts using a semi-quantitative scoring scale (0 (no expression), 1+ (mild expression), 2+ (moderate expression), 3+ (strong expression)). The epithelial and stromal regions of the tissue cores have been evaluated separately. Statistical analysis was performed by using Kruskal-Wallis test and p adjustment was done by Benjamini-Hochberg method. Adjusted p-value of < 0.05 was considered as significant.

### *In silico* DNA Methylation Analysis

DNA methylation status of LINC00152 promoter was analysed using methyl capture sequencing (MethylCap-seq) data of 30 colonic samples [23] (Table 1, Supplementary Table S1). LINC00152-related CpG site (cg00863099) was evaluated on GSE48684 BeadChip450K data set containing the methylation results of 147 colorectal tissues including CRC, AD and NAT samples [24] and on The Cancer Genome Atlas (TCGA) database [25] including CRC and paired NAT tissues. GRCh37/hg19 genomic version was applied for genomic position determination. During statistical evaluation FDR was applied for Student’s t-test with the criteria of p < 0.05 in paired comparisons. Methylation alterations between diagnostic groups were characterised by Δβ-values (the differences of the average β-values of sample groups).

### Bisulfite Sequencing

Methylation status of LINC00152 promoter was analysed on 47 fresh frozen, RNAlater stabilized independent colonic biopsies (Table 1, Supplementary Table S1). Genomic DNA was isolated using High Pure PCR Template Preparation Kit (Roche). Bisulfite conversion was performed using the EZ DNA Methylation Direct Kit (Zymo Research, Irvine, CA, USA). Bisulfite-specific PCR reactions were performed using primers designed with PyroMark Assay Design software (SW 2.0, Qiagen). Primer specificities were tested *in silico* by BiSearch software (http://bisearch.enzim.hu). BS-PCR was performed using LightCycler 480 Probes Master (Roche), LightCycler 480 ResoLight Dye (Roche Diagnostic, Mannheim, Germany), primer mix (200 nM final concentration), bisulfite converted DNA samples (10 ng bcDNA/well) in 15 µl final volume. Real-time PCR amplification was carried out with the following thermocycling conditions on the LightCycler 480 System: 95 °C for 10 min, then 95 °C for 30 sec, 65 °C with 0.5 °C decreasement/cycle for 30 sec, 72 °C for 30 sec for 10 touchdown cycles, followed by the amplification at 95 °C for 30 sec, 60 °C for 30 sec, and 72 °C for 30 sec in 40 cycles. The primer sequences were the following: forward: 5’-GTGTGATTGTTAGGAGGTGTTTT-3’; reverse: 5’-TTATTTTCCAACCAAACACTACAATCTCT-3’; sequencing primer: 5’-GGAGGTGTTTTTTAGTTT-3’. Pyrosequencing was performed on a PyroMark Q24 instrument using PyroMark Gold Q24 Reagents (Qiagen) according to the manufacturer's recommendations. Sequencing results were analysed using the PyroMark Q24 software v2.0.6 (Qiagen).

For statistical evaluation, Student’s t-test/Welch’s test were applied in paired comparisons. In case of evaluation of parallel RNA expression (GSE100179 [22]) and DNA methylation (bisulfite pyrosequencing) data, Pearson correlation was applied due to normal distribution.

### Silencing of LINC00152 in SW480 Colon Carcinoma Cell Line

SW480 colon adenocarcinoma cells from ATCC cultured in L15 medium (Biosera, Nuaille, France) with 2 mM L-glutamine (ThermoFisher-Gibco) supplemented with 10% foetal bovine serum (Biosera), 7.5% sodium bicarbonate (Biosera) and antibiotics (100 IU penicillin, 50 ug/ml streptomycin (GE Healthcare Bio-Sciences Austria, Pasching, Austria) and were maintained in humified air atmosphere at 37°C with 5% CO_2_. For effective silencing of the target gene with high specificity, stabile and less toxic Stealth™ next-generation chemically modified siRNAs [27] were applied as the following: SW480 cells were seeded in 6-well (or 24-well) plates, and 24 hours later were transfected with three different LINC00152 Stealth siRNAs (HSS167198, HSS167199 and HSS167200) (ThermoFisher) simultaneously (si-LINC00152), or Stealth Negative Control Medium GC siRNA (si-NEG) or Stealth GAPDH Positive Control siRNA (ThermoFisher) using Lipofectamine RNAiMax transfection reagent (ThermoFisher-Invitrogen) in 5:1 mix of antibiotics-free L15 medium: Opti-MEM I Reduced Serum Medium (ThermoFisher-Gibco).

After 12 hours, the medium was replaced with fresh 5:1 mix of antibiotics-free L15 and Opti-MEM I medium. Cells were harvested for RT-PCR, FACS, western blot and whole transcriptome microarray analysis 48 or 72 hours after transfection. Silencing efficiency was measured by real-time PCR using LightCycler 480 Probes Master (Roche) and Taqman gene expression assays for LINC00152 (Hs03654334_m1) and GAPDH (Hs02758991_g1) (ThermoFisher). For pairwise comparison, Mann-Whitney test with FDR correction was used and adjusted p-value of < 0.05 was considered as significant.

### Flow Cytometry

Apoptosis was measured by SubG1 analysis prepared as described by Darzynkiewicz *et al*. [28]. 5 × 10^5^ cells were fixed in ice-cold ethanol (70%), followed by alkalic extraction (200 mM Na_2_HPO_2_, pH 7.4), then stained with propidium-iodide (Sigma-Aldrich, St. Louis, MO, USA). Flow cytometric measurements were carried out with FACSCalibur (BD Biosciences) and analysed by Kaluza software (Beckman Coulter). Displayed results are geometric mean fluorescence intensities (MFI) normalized to the percentage of apoptotic cells by apoptosis measurements. For pairwise comparison, Mann-Whitney test with FDR correction was used and adjusted p-value of < 0.05 was considered as significant.

### Genome-wide Expression Analysis in LINC00152-silenced SW480 Cells

After total RNA isolation from the harvested SW480 cells by Qiagen RNeasy Mini kit (Qiagen), HTA2.0 microarray analysis was performed from 100 ng of DNase-treated total RNA samples (3–3 parallels from si-LINC00152 and si-NEG cells, 72 hours after transfection) according to the manufacturer’s instructions. The dataset was uploaded to the GEO database with GSE111413 accession number. Quality control and pre-processing with SST-RMA, then gene level analysis were performed using Transcriptome Analysis Console 4.0 (TAC4.0) (ThermoFisher-Affymetrix). DEGs between si-LINC00152 and si-NEG samples were determined by using paired Student's t-test with FDR correction (adjusted p-value of < 0.05 was considered as significant). For LogFC calculation, the differences between the averages of sample groups were considered with abs ≥ 1 criteria. In order to expand the number of classifiable DEGs, integrated, non-automatic pathway analysis was performed using several pathway analysis databases including The Database for Annotation, Visualization and Integrated Discovery v6.8, TAC4.0 using WikiPathways, NCBI Biosystems Database using KEGG, BioCyc, Reactome, National Cancer Institute's Pathway Interaction Database, WikiPathways, and Gene Ontology records and literature data were also involved.

### Western Blot Analysis

For Western blot analysis protein extracts from lysed cells were quantified using Bradford reagent (BioRad, Hercules, CA, USA) and were separated by SDS-PAGE. Proteins were transferred to PVDF membrane applying semidry technique (BioRad), membranes were incubated with the following antibodies: rabbit polyclonal anti-PORCN antibody (1:500, Abcam, Cambridge, UK), rabbit monoclonal anti-YES1 antibody (1:1000; Abcam), rabbit monoclonal anti-cyclin D1 antibody (SP4, 1:500, ThermoFisher-Invitrogen) and rabbit monoclonal anti-phospho-S6 ribosomal protein antibody (1:1000, Cell Signaling Technology, Leiden, The Netherlands) and mouse monoclonal anti-β-actin antibody (1:5000; Sigma-Aldrich) as loading control. Finally, biotinylated secondary antibodies, avidin-HRP complex (Vectastain Elite ABC Kit, Vector Laboratories, Burlingame, CA, USA) and enhanced chemiluminescence technique (Pierce ECL Western Blotting Substrate (ThermoFisher) using C-Digit western blot detection system (Li-Cor Biotechnology, Lincoln, NE, USA) were applied.

## Results

### LINC00152 Expression in Colorectal Tissue Samples

**Fig. 1:**
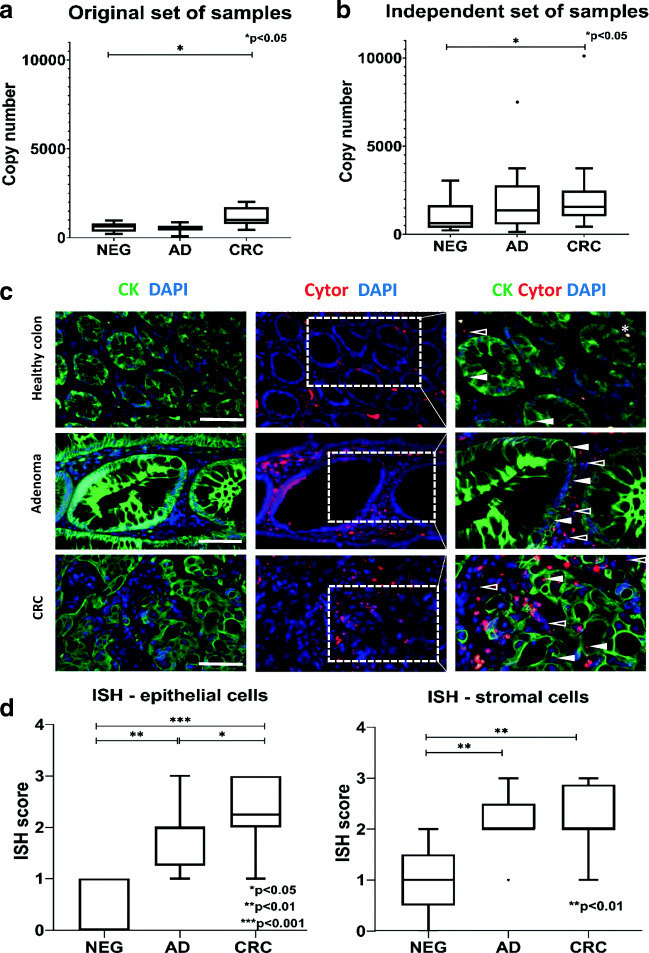
Validation of LINC00152 expression in colonic biopsy samples by real-time PCR and ***in situ*** hybridization. **A**. LINC00152 was significantly up-regulated in CRC vs. normal samples, and in CRC vs. adenoma samples (p < 0.02), when the original sample set was analysed. NEG = healthy normal (n = 10), AD = adenoma (n = 10), CRC = colorectal cancer (n = 10). **B**. Significant overexpression of LINC0052 could be verified in CRC biopsy specimens compared to normals on an independent set of samples (p < 0.017). NEG = healthy normal (n = 20), AD = adenoma (n = 20), CRC = colorectal cancer (n = 20). **C**. Representative LINC00152 *in situ* hybridization (CYTOR, red signals) images of normal, adenoma and CRC FFPE tissue sections (cell nuclei are stained blue with DAPI). LINC00152 was found to be up-regulated both in the cytokeratin (CK) positive (green immunofluorescence) epithelial cells (full arrowheads) and stromal cells (empty arrowheads) of adenoma and more intensely of CRC samples compared to healthy normal colon tissue (merged images, right column). The scale bar represents 150 μm on the left and middle columns, and 100 μm on the right column of magnified insets. **D**. *In situ* hybridization analysis on tissue microarrays revealed significant up-regulation of LINC00152 expression in colorectal tumours both in epithelial and stromal cells (*p < 0.05; **p < 0.01; ***p < 0.001). NEG = healthy normal (n = 5), AD = adenoma (n = 29), CRC = colorectal cancer (n = 20).

Our *in silico* LINC00152 expression results detected on Affymetrix HGU133Plus2.0 and Human Transcriptome Array 2.0 microarrays were in accordance with literature data about the up-regulation of LINC00152 in the colorectal carcinogenesis (Supplementary Fig. 1A and 1B). Significant overexpression of LINC00152 in CRC samples was confirmed by real-time PCR both on 30 original (10 normals, 10 adenomas and 10 CRCs) and on the independent set of 60 (20 normals, 20 adenomas and 20 CRCs) samples (original CRC vs. N samples: adjusted p < 0.02 (Mann-Whitney test with FDR correction) Fig. 1A; independent CRC vs. N samples: adjusted p < 0.017 (Mann-Whitney test with FDR correction) Fig. 1B).

### *In situ* Hybridization

As *in silico* analysis of a HGU133 Plus2.0 microarray data set (GSE15960 [29]) containing expression data from laser capture microdissected (LCM) colonic epithelial and stromal cells from normal, adenoma and CRC tissues resulted in siginificant overexpression of LINC00152 in both cell types in CRC (Supplementary Fig. 1C), for deeper insight, we aimed to explore the *in situ* expression and localization of LINC00152 in the colorectal normal-adenoma-CRC sequence using ISH on TMA slides (containing tissue cores from 5 normal, 29 adenoma and 20 CRC tissue samples).

According to the ISH results, scarce cytoplasmic LINC00152 RNA signals, observed in healthy colonic mucosa, showed gradual increase both in the epithelial and stromal compartments from adenoma to carcinoma progression (Fig. 1C). The statistical analysis of the TMA ISH results revelaed elevated LINC00152 expression in colorectal tumours both in epithelial and stromal cells (epithelial region: p = 0.0030 (AD vs. N), p = 0.0001 (CRC vs. N); stromal region: p = 0.0018 (AD vs. N), p = 0.0015 (CRC vs. N)) (Fig. 1D).

### Promoter Methylation Status of LINC00152

Using methyl capture sequencing data [23] (6 NAT, 15 adenoma and 9 CRC samples), significant hypomethylation of predicted LINC00152 promoter was determined in CRC compared to NAT samples (p = 0.0011). Decreased, but not significant methylation alteration was also detected in AD vs. NAT and CRC vs. AD comparisons. *In silico* analysis of BeadChip450K methylation data of Luo *et al*. [24] (41 NAT/normal, 42 adenoma and 64 CRC samples) revealed highly significant hypomethylation of cg00863099 CpG site within LINC00152 promoter in CRC samples compared to N/NAT groups (p < 0.0001). DNA methylation level of adenoma samples was higher than of CRCs, but lower than N/NAT samples. Data set of TCGA [25] (38 NAT and 301 CRC samples) also showed remarkable hypomethylation in CRCs compared to NAT samples. Table 2 summarizes the *in silico* methylation analysis results of LINC00152 promoter region in colorectal tissue samples.

**Table 2:** Summary of *in silico* DNA methylation data of LINC00152 promoter

	Methyl capture sequencing [23]			Illumina BeadChip 450K (Luo *et al*.) [24]					Illumina BeadChip 450K (TCGA) [25]
Analysed region	chr2: 87.754.801–87.754.900 between TSS-173 and TSS-73			cg00863099 (chr2: 87.755.372)TSS + 398					
Comparisons	CRC vs. NAT	AD vs. NAT	CRC vs. AD	CRC vs. NAT	CRC vs. N	AD vs. NAT	AD vs. N	CRC vs. AD	CRC vs. NAT
p-value	1.1 × 10^− 3^	1.0 × 10^− 1^	1.3 × 10^− 1^	2.17 × 10 ^− 10^	3.27 × 10 ^− 12^	3.7 × 10^− 1^	1.4 × 10^− 2^	3.93 × 10 ^− 13^	
Δβ-value	-0.31	-0.18	-0.13	-0.38	-0.44	-0.044	-0.11	-0.33	-0.56

When significant DNA methylation differences (p < 0.05) were found, the given comparison was underlined. CRC = colorectal cancer; AD = adenoma; NAT = normal adjacent tissue; N = healthy normal. Methyl capture sequencing study included 30 samples (6 NAT, 15 AD and 9 CRC), Illumina BeadChip 450K study of Luo *et al*. contains methylation data from 147 samples (41 N/NAT, 42 AD and 64 CRC), and 339 samples (38 NAT and 301 CRC) were involved in the Illumina BeadChip 450K project of TCGA.

**Fig. 2:**
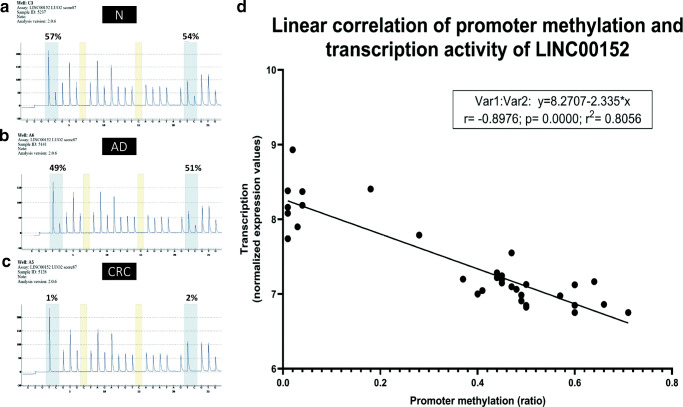
Bisulfite sequencing analysis results of LINC00152 promoter and parallel LINC00152 expression data. Representative LINC00152 bisulfite sequencing results in normal (A), adenoma (B) and CRC (C) tissue samples and parallel LINC00152 expression and promoter DNA methylation data (D). **A**. Normal samples showed significant hypermethylation compared to CRC samples (48 ± 7% for CpG1 and 48 ± 6% for CpG2). **B**. Methylation percentages were intermediate in adenoma samples, but they were closer to percentages measured in normal controls (44 ± 16% for CpG1 and 43 ± 15% for CpG2). **C**. In CRC samples the LINC00152 promoter region was strongly hypomethylated with 4 ± 8% average methylation percentage for CpG1 site, and with 5 ± 6% for CpG2 site. CpG1 site is represented on Illumina BeadChip450K with cg00863099 ID. N = healthy normal, AD = adenoma, CRC = colorectal cancer **D**. Strong negative correlation between promoter DNA methylation and expression of LINC00152 (r=-0.8976) was found according to the parallel expression and promoter methylation analysis results of the same set of colonic biopsy specimen (11 normal, 13 adenoma and 9 CRC samples).

LINC00152 promoter methylation status was verified using bisulfite sequencing of DNA from 12 CRC, 22 adenoma and 13 normal biopsy samples. In CRC cases significantly reduced LINC00152 promoter DNA methylation was measured compared to normal (CpG1: p = 1.1 × 10^− 13^, Δmet%= -44%; CpG2: p = 1.8 × 10^− 14^, Δmet%= -43%) and adenoma samples (CpG1: p = 2.9 × 10^− 11^, Δmet%= -40%; CpG2: p = 9.8 × 10^− 12^, Δmet%= -39%). In adenoma samples lower DNA methylation levels, but not significant LINC00152 promoter DNA methylation changes were detected compared to normal samples. Representative bisulfite pyrosequencing sequenograms are shown in Fig. 2A, B and C.

### Parallel Analysis of Promoter Methylation and LINC00152 Expression

Parallel RNA expression (HTA2.0, GSE100179 [22]) and DNA methylation data (bisulfite pyrosequencing) from the same sample set containing 11 normal, 13 adenoma and 9 CRC biopsies revealed strong negative correlation between DNA methylation and expression of LINC00152 showing overexpression and hypomethylation in CRC (r=-0.8976, r^2^ = 0.8056) (Fig. 2D).

### The Effects of LINC00152 Silencing on Cell Cycle in SW480 Colon Carcinoma Cells

**Fig. 3:**
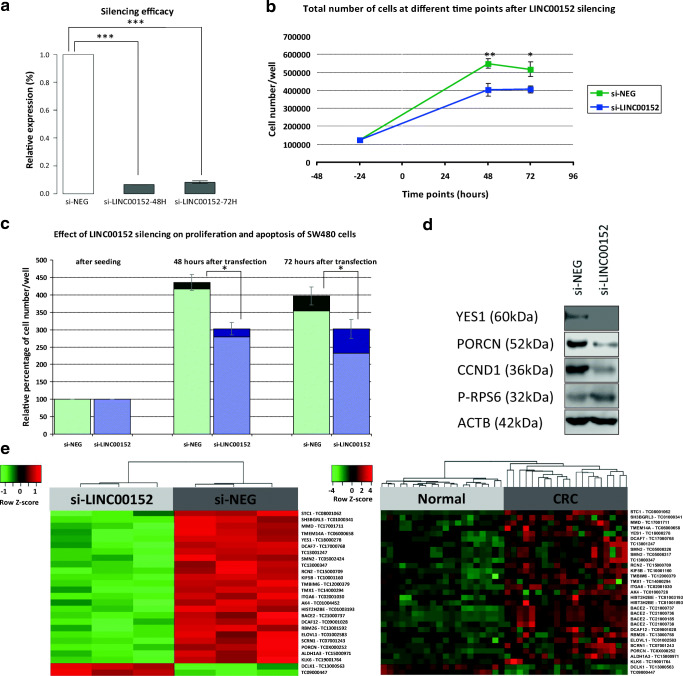
Effects of LINC00152 silencing on cell proliferation, apoptosis and the expression of cyclin D1, phosphorylated ribosomal S6, YES1 and PORCN proteins in SW480 colon carcinoma cells. **A**. Relative LINC00152 expression in SW480 cells transfected with negative control (si-NEG) or LINC00152 siRNAs, 48 hours (si-LINC00152 48H) (average values from 3 transfections) and 72 hours (si-LINC00152 72H) (average values from 4 transfections) after treatment, respectively, calculated according to the quantitative RT-PCR results. The residual LINC00152 expression under 10% refers to silencing efficiency over 90% (*** = p < 0.001, Mann-Whitney test). **B**. Total number of SW480 cells transfected with negative control (si-NEG) or LINC00152 siRNAs (si-LINC00152) at different time points (3–3 parallel measurements). LINC00152 silencing significantly inhibited the proliferation of SW480 cells both 48 hours and 72 hours after treatment (* = p < 0.05, ** = p < 0.01, Mann-Whitney test). Transfection was performed at ‘0 hours’ time point, and the seeding of cells in 6-well plates was carried out 24 hours before transfection (at ‘-24 hours’ time point). **C**. Effect of LINC00152 silencing on proliferation and apoptosis of SW480 cells (according to 3–3 parallel measurements). The columns represent the percentages of viable (based on trypan blue exclusion) cell numbers compared to cell numbers after seeding in six well plates (‘-24 hours’ = 24 hours before transfection: 125 000 cells = 100%). The part of the column with light color means the rate of proliferating cells, while the darker part represents the percentage of apoptotic cells. The standard deviations of percentages of viable cell numbers are represented. The LINC00152 silencing significantly reduced the proliferation and induced the apoptosis of SW480 cells (* = p < 0.05, Mann-Whitney test). **D**. Representative western blot images of cyclin D1 (CCND1), phosphorylated S6 ribosomal protein (P-RPS6), YES proto-oncogene 1, Src family tyrosine kinase (YES1) and porcupine homolog (Drosphila) (PORCN) proteins. As a loading control beta-actin (ACTB) was used. Reduced cyclin D1 protein level was detected after 72-hour incubation in the LINC00152 siRNA-treated cells without attenuation of the mTOR activity indicator phospho-S6 ribosomal protein expression. Similarly to mRNA expression changes, decreased expression of PORCN and YES1 WNT signalling pathway associated proteins was found in LINC00152- silenced cells. The cropped blot images are shown in Fig. 3D and the full-length blots are presented as Supplementary Fig. 2. **E**. Reversal effect of the LINC00152 silencing on the expression of genes deregulated in CRC. Heatmap of significantly differentially expressed genes (DEGs) in LINC00152-silenced SW480 cells compared to negative control cells (3–3 paralells) which expressed reversely in CRC vs. normal comparison (adjusted p < 0.05, right). Overexpression is marked with red, while down-regulated genes are green. si-LINC00152 = SW480 cells transfected with LINC00152 siRNAs; si-NEG = SW480 cells transfected with negative control si-RNA. Heatmap of significantly DEGs in CRC and normal tissue biopsy samples (20 normal and 20 CRC samples) showing inverse expression in si-LINC00152 vs. si-NEG comparison (adjusted p < 0.05, left). Up-regulated genes are marked with red, while underexpressed genes are green. CRC = colorectal cancer

LINC00152 expression was successfully silenced in SW480 cells with 90–93% efficiency (according to average values from 3 and 4 parallel transfections, respectively) (Fig. 3A). Silencing of LINC00152 significantly suppressed cell growth compared to negative control cells both 48 and 72 hours after transfection (3–3 parallel measurements) (p < 0.05) (Fig. 3B and C).

LINC00152 silencing induced apoptosis, as well. Percentage of apoptotic cells in SW480 cells transfected with LINC00152 siRNAs was higher than in negative control: both after 48 and 72 hours, LINC00152 knockdown caused approximately two-fold increase in apoptosis (48H: si-NEG: 4% and si-LINC00152: 8%; 72H: si-NEG: 11% and si-LINC00152: 23%) (Fig. 3C).

In parallel with the growth inhibitory effect, silencing of LINC00152 could reduce cyclin D1 expression. Western blot analysis resulted in cyclin D1 protein level decrease after 72-hour incubation in the LINC00152 siRNA-treated cells without attenuation of the mTOR activity indicator phospho-S6 ribosomal protein expression (Fig. 3D).

### Molecular Pathways Influenced by LINC00152 Silencing According to the Whole Transcriptome Analysis Results

On the basis of HTA2.0 microarray results, 247 genes (149 down-regulated and 68 overexpressed) were identified showing significantly altered expression 72 hours after LINC00152 silencing (adjusted p < 0.05). Using the absolute value of fold change ≥ 2 criteria, the list of DEGs was shortened to 74 (44 underexpressed and 30 up-regulated genes) (Table 3). Among these DEGs several genes with oncogenic and/or metastasis promoting function (e.g. STC1, YES1, PORCN) were found to be down-regulated and certain tumour suppressor genes (e.g. DKK1, PERP) showed overexpression in LINC00152-silenced cells. The reduced expression of PORCN and YES1 WNT signalling pathway-associated molecules in LINC00152-silenced cells was also confirmed at protein levels (Fig. 3D). The representative Western blot images are presented in Fig. 3D and the full-length blots are attached as Supplementary Fig. 2. Twenty percentage of DEGs could not be associated with annotated transcripts.

**Table 3:** The top differentially expressed genes in LINC00152 silenced SW480 colon carcinoma cells

HTA 2.0 ID	Gene Symbol	Gene name	Fold Change	Adjusted p-value
TC02003499.hg.1			-6.40	0.0075
TC08001062.hg.1	STC1	stanniocalcin 1	-3.51	0.045
TC01002441.hg.1	LAPTM5	lysosomal protein transmembrane 5	-3.40	0.039
TC01001090.hg.1	TXNIP	thioredoxin interacting protein	-3.17	0.029
TC11001031.hg.1			-3.17	0.048
TC09000799.hg.1	PAEP	progestagen associated endometrial protein	-3.13	0.021
TC01000341.hg.1	SH3BGRL3	SH3 domain binding glutamate rich protein like 3	-3.11	0.019
TC17001711.hg.1	MMD	monocyte to macrophage differentiation associated	-2.96	0.024
TC02003706.hg.1	MTX2	metaxin 2	-2.94	0.022
TC06000658.hg.1	TMEM14A	transmembrane protein 14A	-2.92	0.029
TC18000278.hg.1	YES1	YES proto-oncogene 1, Src family tyrosine kinase	-2.86	0.021
TC03003299.hg.1			-2.74	0.013
TC17000768.hg.1	DCAF7	DDB1 and CUL4 associated factor 7	-2.71	0.025
TC13001247.hg.1			-2.70	0.0075
TC17002458.hg.1	DERL2	derlin 2	-2.65	0.031
TC16001407.hg.1			-2.62	0.045
TC03001071.hg.1	HES1	hes family bHLH transcription factor 1	-2.52	0.024
TC11002316.hg.1	CADM1	cell adhesion molecule 1	-2.51	0.0075
TC05002424.hg.1	SMN2	survival of motor neuron 2, centromeric	-2.50	0.033
TC16000151.hg.1	C16orf72	chromosome 16 open reading frame 72	-2.47	0.047
TC13000347.hg.1			-2.43	0.028
TC13000100.hg.1	ALOX5AP	arachidonate 5-lipoxygenase activating protein	-2.42	0.023
TC15000709.hg.1	RCN2	reticulocalbin 2	-2.42	0.023
TC10001160.hg.1	KIF5B	kinesin family member 5B	-2.41	0.0075
TC08001199.hg.1	SNAI2	snail family transcriptional repressor 2	-2.33	0.021
TC12000379.hg.1	TMBIM6	transmembrane BAX inhibitor motif containing 6	-2.32	0.013
TC14000294.hg.1	TMX1	thioredoxin related transmembrane protein 1	-2.31	0.028
TC02001030.hg.1	ITGA6	integrin subunit alpha 6	-2.27	0.019
TC11003188.hg.1			-2.27	0.022
TC02001057.hg.1	MTX2	metaxin 2	-2.18	0.042
TC11000628.hg.1	NEAT1; MIR612	nuclear paraspeckle assembly transcript 1; microRNA 612	-2.17	0.039
TC01004452.hg.1	AK4	adenylate kinase 4	-2.16	0.048
TC01003193.hg.1	H2BC21 (HIST2H2BE)	H2B clustered histone 21	-2.15	0.024
TC21000737.hg.1	BACE2	beta-secretase 2	-2.13	0.031
TC09001028.hg.1	DCAF12	DDB1 and CUL4 associated factor 12	-2.12	0.025
TC02001074.hg.1	PLEKHA3	pleckstrin homology domain containing A3	-2.09	0.035
TC13001592.hg.1	RBM26	RNA binding motif protein 26	-2.09	0.029
TC01002583.hg.1	ELOVL1	ELOVL fatty acid elongase 1	-2.08	0.031
TC07001243.hg.1	SCRN1	secernin 1	-2.08	0.028
TC0 × 000252.hg.1	PORCN	porcupine O-acyltransferase	-2.07	0.028
TC06002263.hg.1	DYNLT1	dynein light chain Tctex-type 1	-2.03	0.028
TC15000971.hg.1	ALDH1A3	aldehyde dehydrogenase 1 family member A3	-2.01	0.029
TC19001764.hg.1	KLK6	kallikrein related peptidase 6	-2.01	0.029
TC01000408.hg.1	SERINC2	serine incorporator 2	-2.01	0.028
TC06002157.hg.1	PERP	p53 apoptosis effector related to PMP22	2.00	0.043
TC01005497.hg.1	NDC1	NDC1 transmembrane nucleoporin	2.02	0.031
TC02003847.hg.1			2.02	0.021
TC20000603.hg.1	FERMT1	fermitin family member 1	2.06	0.028
TC02000521.hg.1	SNORD94	small nucleolar RNA, C/D box 94	2.11	0.045
TC12001216.hg.1	OLR1	oxidized low density lipoprotein receptor 1	2.13	0.028
TC04001824.hg.1	FAT1	FAT atypical cadherin 1	2.16	0.028
TC05003439.hg.1	ITGA1; PELO	integrin subunit alpha 1; pelota mRNA surveillance and ribosome rescue factor	2.18	0.0096
TC09000335.hg.1	ANXA1	annexin A1	2.21	0.035
TC07001808.hg.1	FAM3C	family with sequence similarity 3 member C	2.32	0.012
TC13000563.hg.1	DCLK1	doublecortin like kinase 1	2.36	0.028
TC11002587.hg.1	SAA1	serum amyloid A1	2.37	0.032
TC09000677.hg.1	LCN2	lipocalin 2	2.39	0.019
TC02000198.hg.1			2.43	0.045
TC05000550.hg.1	ARL14EPL	ADP ribosylation factor like GTPase 14 effector protein like	2.44	0.042
TC02001364.hg.1	CCL20	C-C motif chemokine ligand 20	2.53	0.020
TC04000408.hg.1	CXCL8	C-X-C motif chemokine ligand 8	2.69	0.044
TC05002579.hg.1			2.76	0.035
TC10000350.hg.1	DKK1	dickkopf WNT signalling pathway inhibitor 1	2.82	0.035
TC02002747.hg.1	FN1	fibronectin 1	2.82	0.0075
TC05002512.hg.1	NR2F1	nuclear receptor subfamily 2 group F member 1	2.86	0.035
TC0 × 001623.hg.1			2.90	0.0075
TC09000447.hg.1			2.95	0.019
TC05001352.hg.1	ESM1	endothelial cell specific molecule 1	3.04	0.031
TC20000341.hg.1	PI3	peptidase inhibitor 3	3.05	0.021
TC02003848.hg.1			3.11	0.038
TC04002067.hg.1	CXCL8	C-X-C motif chemokine ligand 8	3.40	0.035
TC16000401.hg.1			3.90	0.028
TC05003120.hg.1			4.94	0.029
TC05001550.hg.1	LOC105379051	uncharacterized LOC105379051	5.86	0.021

The table contains average fold change data of Human Transcritome Array 2.0 expression values from 3–3 parallels from LINC00152 silenced vs. negative control SW480 colon adenocarcinoma cells, 72 hours after transfection.

Integrated pathway analysis of 247 DEGs revealed that PI3K/Akt, Ras, WNT, TP53, Notch and ErbB signalling pathways were highly involved, while the mTOR pathway did not appear to be affected as none of the identified DEGs could be assigned to mTOR signalling cascade (Supplementary Table S2).

### Reversal Effect of LINC00152 Silencing on the Expression of Genes Deregulated in CRC

The mRNA expression levels of 74 DEGs identified in LINC00152-silenced cells (3–3 paralells) were also analysed *in silico* using whole transcriptome data of colonic tissue samples (GSE100179 [22], 20 normal and 20 CRC samples). The expression of 26 out of the 74 genes (35.14%) changed oppositely after LINC00152 silencing compared to their characteristic expression in CRC vs. normal tissue (adjusted p < 0.05) indicating that the LINC00152 silencing has a reversal effect on the expression of several genes deregulated in CRC including overexpressed *PORCN*, *KLK6*, *YES1* proto-oncogene, *ITGA6* and *STC1* pro-metastatic genes (Fig. 3E) which were found to be down-regulated in LINC00152-silenced SW480 cells.

## Discussion

LncRNAs are important epigenetic regulatory molecules contributing to the development of different cancers including gastrointestinal cancers. LINC00152 (also known as CYTOR) is one of the most extensively investigated lncRNAs in GC, but its expression, promoter DNA methylation and effects during colorectal normal-adenoma-carcinoma sequence progression have not been completely studied yet. Cell culture experiments revealed that LINC00152 exerts its oncogenic function through facilitation of cell proliferation via mTOR [9], PI3K/Akt [15, 30], mir193-3p/ERBB4/Akt [17] and miR139-5p/NOTCH1 [16] signalling pathways or via participating in epigenetic silencing of p15, p21 [8] and p16 [31] tumour suppressor genes. Microarray-based study of Ji *et al*. revealed the involvement of mTOR signalling pathway in HCC [9]. However, the complex network of molecular pathways affected by LINC00152 has not been studied in CRC yet.

In this research, we studied the *in situ* expression of LINC00152 during the colorectal normal-adenoma-carcinoma sequence progression and analysed the potentially underlying DNA methylation alterations on its promoter region. The effects of LINC00152 on cell growth and its possible gene expression regulatory role were also investigated at whole transcriptome level in SW480 colon carcinoma cell line.

Similarly to the previous findings in gastrointestinal [6–13, 15–18, 30, 32–34] and other cancers [31, 35, 36], elevated LINC00152 lncRNA levels were detected in CRC tissue samples in our study both by microarray and by RT-PCR analyses. Previous *in silico* evaluations (Supplementary Fig. 1, and furthermore, GSE18105 [37]: p < 0.0001, logFC = 0.91; GSE9348 [38]: p < 0.0001, logFC = 1.42) also supported its up-regulation in CRC compared to normal controls. However, it must be noted that Zhang YH *et al*. [39] – in contrast to the results of other research groups [16–18, 40–42] - published underexpression of LINC00152 in CRC [39]. Our *in situ* hybridization results in colorectal surgical FFPE tissue sections and TMA slides revealed significant up-regulation of LINC00152 through the normal-adenoma-carcinoma sequence progression, both in epithelial and stromal cells (Fig. 1C and D). As far as we are aware, this is the first time that LINC00152 is detected in colorectal tissues *in situ*.

Overexpression of LINC00152 is reported to be associated with tumour progression and shorter survival in GC [10, 11], HCC [9], renal cell carcinoma [35], gallbladder cancer [30] and also with shorter overall and recurrence-free survival in CRC [16, 17]. In our study, relationship between the degree of LINC00152 up-regulation and clinical parameters of CRC and adenoma samples (such as tumour stage, location, histological types of adenoma, severity of dysplasia) could not be detected. For studying the possible relationship between the molecular features of CRC and the LINC00152 expression, microarray data of Marisa *et al*. (GSE39582 [43]) was *in silico* analysed. The results refer to that CIMP, MSI and BRAF mutation status of CRC may slightly correlate with LINC00152 expression, while no association was found between TP53 and KRAS mutation and LINC00152 expression levels (Supplementary Table S3).

Promoter DNA methylation changes behind the LINC00152 expression alterations were also examined. To the best of our knowledge, we are the first to demonstrate significant hypomethylation of probable LINC00152 promoter region in CRC compared to normal controls. In CRC, hypomethylation in two different regions within LINC00152 promoter were detected: between transcription start site (TSS)-173 and TSS-73 using MethylCap-seq and between TTS + 396 and TSS + 501 using bisulfite sequencing. The first region was considered as an active promoter only one out of the nine analysed cell lines according to the Encode ChromHMM data [44], while the second region was defined as ’active promoter’ in 8 out of 9 cell lines [44]. The latter region (TSS + 396–TSS + 501) includes the CpG site represented on BeadChip450K array with cg00863099 (chr2:87.755.372), which we also found strongly hypomethylated in CRC during *in silico* analysis of data sets of Luo *et al*. [24] and of TCGA [25]. Similarly to our results in CRC, significant hypomethylation of LINC00152 promoter was found in HCC [14].

Using parallel RNA expression and DNA methylation data, strong negative correlation was revealed between DNA methylation and expression of LINC00152 suggesting that transcription of LINC00152 can be regulated by DNA methylation. Gene silencing through DNA hypermethylation of CpG sites within CpG islands in gene promoters is a well-characterised phenomenon, but - although the promoter regions of almost half of human genes do not consist CpG islands - the effect of DNA methylation in non-CpG island promoters on gene expression regulation is not well described [45, 46]. With bioinformatic evaluation of genome-wide transcriptome and methylome data, a strong association was found between transcription and the number of unmethylated CpGs at non-CpG island promoters [47]. Experimental results of others support the above regulatory role: DNA methylation directly silenced the transcription of genes with non-CpG island promoters [45], in other words inverse relationship between promoter methylation and gene expression - similarly to the CpG island containing promoters - is also characteristic for non-CpG island promoters [45, 46]. Moreover, Oster *et al*. have concluded that ‘hypomethylation of non-CpG island promoters deregulate gene expression nearly as frequently as does the hypermethylation of CpG island promoters’ [46].

Besides the detection of promoter DNA methylation changes, in order to explore the role of copy number variation (CNV) of LINC00152 gene as a potential phenomenon behind the LINC00152 expression alterations in colorectal tumours, we have performed parallel analyses of LINC00152 expression, promoter DNA methylation and CNV of its coding gene on GDC TCGA COAD (Colon Adenocarcinoma) data set. Our results, in line with the i*n silico* results, suggest that promoter hypomethylation of LINC00152 can be the primary mechanism leading to its overexpression in CRC, while CNV might have only a minor role in this (Supplementary Fig. 3.).

Accordingly, our results show that hypomethylation of LINC00152 promoter is associated with its increased expression in CRC suggesting a functional role of LINC00152 in colorectal carcinogenesis by the loss of promoter methylation. Therefore, we investigated the effect of LINC00152 on cell cycle regulation since un-controlled cell division is a key factor in malignant transformation. In accordance with the oncogenic role of LINC00152 in different cancer types [8, 9, 11, 15–17, 30, 31, 48], the results of our silencing experiments indicate that this lncRNA promotes cell proliferation and suppresses apoptosis in SW480 cells, as well. The oncogenic function of LINC00152 is also supported by our whole transcriptome analysis results of LINC00152-silenced SW480 cells showing that LINC00152 silencing has a reversal effect on the expression of several genes deregulated in CRC. LINC00152 knockdown decreased cyclin D1 protein expression without attenuation of mTOR activity according to the phospho-S6 ribosomal protein expression data. In contrast to the involvement of mTOR signalling observed in HCC cell lines [9], our results suggest that LINC00152 may influence the cyclin D1 expression through other signal transduction pathways in CRC.

For the comprehensive analysis of affected molecular pathways, we performed whole transcriptome microarray analysis after LINC00152 silencing. The pathway analysis also supported the participation of a wide range of molecular pathways including PI3K/Akt, Ras, WNT, TP53, Notch and ErbB, but not the mTOR signalling in the mechanism of cell cycle facilitating action of LINC00152. Accordingly, the involvement of PI3K/Akt, ErbB and Notch pathways has been previously reported in different types of cancers [15–17, 49]. Whole transcriptome microarray analysis of LINC00152-silenced cells revealed significant underexpression of genes with oncogenic and/or metastasis promoting function (e.g. *STC1*, *YES1*, *PORCN*) and up-regulation of tumour suppressor genes (e.g. *DKK1*, *PERP*). Among these DEGs, certain genes are important regulatory molecules of WNT and TP53 signalling pathways. YES1 (YES proto-oncogene 1, Src family tyrosine kinase) contributes to the assembly of YAP1-TBX5-beta-catenin transcription factor complex binding to the promoters of anti-apoptotic genes via phosphorylation of YAP1 [50]. Inhibition of YES1 – that was found to be down-regulated after LINC00152 silencing both at mRNA and protein levels – was reported to lead to reduced cell proliferation of beta-catenin-dependent cancers [50]. Porcupine O-acyltransferase (PORCN) post-transcriptionally palmitoleates WNTs which is essential for their secretion and binding to Frizzled receptors [51]. Not only the canonical, but all types of WNT pathways can be inhibited with blocking of PORCN [51]. LINC00152 knockdown down-regulated its mRNA and protein expression supporting the hypothesis that LINC00152 is an important regulatory molecule contributing to the constitutive activation of WNT pathway in CRC. Moreover, LINC00152 silencing resulted in the overexpression of dickkopf WNT signalling pathway inhibitor 1 (*DKK1*). Our LINC00152 silencing experiments suggest that disturbance of TP53 signalling pathway can also be involved in increased apoptosis rate and reduced cyclin D1 expression caused by LINC00152 knockdown, as – among others – *PERP* TP53 apoptosis effector gene was found to be up-regulated in LINC00152-silenced CRC cells. The loss of this potential tumour suppressor gene promotes carcinogenesis by enhancing cell survival, desmosome loss and inflammation [52].

In summary, up-regulation of LINC00152 lncRNA was detected in CRC tissue. Using bisulfite sequencing, to the best of our knowledge, we are the first to demonstrate significant hypomethylation of LINC00152 promoter region in CRC compared to normal controls which was validated on a large independent set of samples. Parallel RNA expression and DNA methylation data revealed that transcription of LINC00152 lncRNA can be regulated by DNA methylation. Promoter hypomethylation and overexpression of LINC00152 can contribute to CRC pathogenesis facilitating cell cycle progression through multiple molecular pathways including WNT, Notch and TP53 signalling pathways. Our results support that overexpression of LINC00152 in CRC can be one of the important causative oncogenic factors leading to malignant phenotype. As up-regulation of LINC00152 plays a role in cancer-related signalling pathways and is often associated with aggressive cancer types, it is expected to become an effective molecular marker for CRC diagnosis and prognosis [48].

## Electronic supplementary material

ESM 1 (PDF 348 kb)

## Data Availability

The data that support the findings of this study are available in Gene Expression Omnibus database at https://www.ncbi.nlm.nih.gov/geo/, reference number GSE111413. These data were derived from the following resources available in the public domain: https://www.ncbi.nlm.nih.gov/geo/
