# Supplementary material for: Promoter Hypomethylation and Increased Expression of the Long Non-coding RNA LINC00152 Support Colorectal Carcinogenesis
Source: Pathol Oncol Res. 2020 Apr 20;26(4):2209–23. doi: 10.1007/s12253-020-00800-8 (PMC7471146; doi:10.1007/s12253-020-00800-8)
Supplement: Supplementary file 1 — (PDF 348 kb) [file 12253_2020_800_MOESM1_ESM.pdf]

Supplementary Figure 1. LINC00152 expression in normal, adenoma and colorectal cancer tissue samples – *in silico* analysis results of microarray data

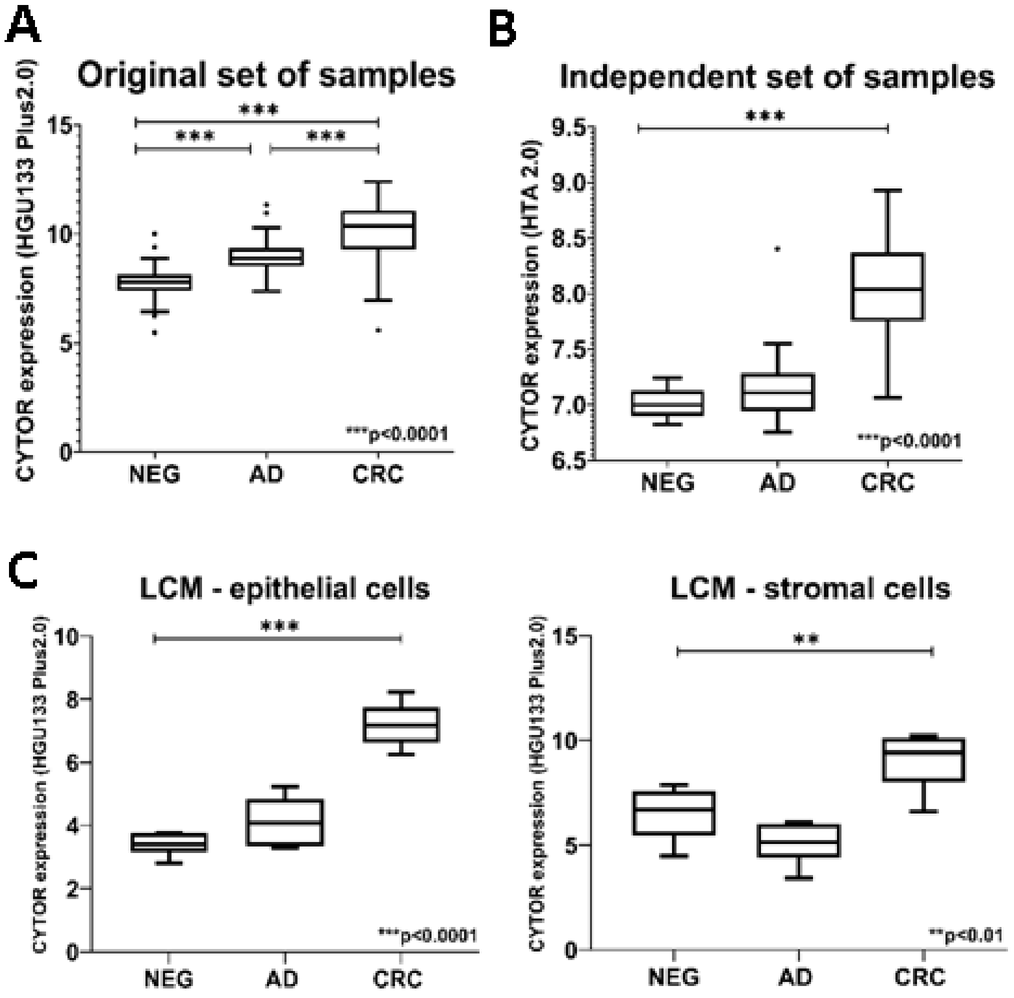

A. On HGU133Plus2.0 microarrays (GSE4183 [19], GSE10714 [20] and GSE37364 [21]), LINC00152 transcript (Affymetrix ID 225799_at) was found to be significantly up-regulated in AD (n=49) and CRC (n=49) biopsy samples compared to healthy controls (n=49), and this overexpression was more intensive in cancer samples (p<0.0001). Significant up-regulation of LINC00152 was detected in CRC samples compared to adenoma tissue, as well (p<0.0001). LINC00152 expression was associated with neither tumour stage, nor tumour location. There was no difference in LINC00152 levels between the patient groups according to the histological type of adenomas, and the severity of dysplasia. **B.** HTA2.0 data (GSE100179 [22]) also supported the overexpression of LINC00152 in CRC (n=20) compared to normal (n=20) biopsy samples (probe set ID: TC02003382.hg.1; p<0.0001). **C.** HGU133Pls2.0 microarray data set (GSE15960 [29]) containing expression data from laser capture microdissected (LCM) colonic epithelial and stromal cells from normal (n=6 epithelial;6 stromal), adenoma (n=6;6) and CRC (n=6;6) tissues. These data also revealed the upregulation of LINC00152 (Affymetrix ID 225799_at) in CRC in both epithelial and stromal compartments (epithelial cells - CRC vs. N: p <0.0001; stromal cells – CRC vs. N: p<0.01).

Supplementary Figure 2.

Full-length western blot images representing the effects of LINC00152 silencing on the expression of YES1, cyclin D1, PORCN and phosphorylated ribosomal S6 proteins in SW480 colon carcinoma cells

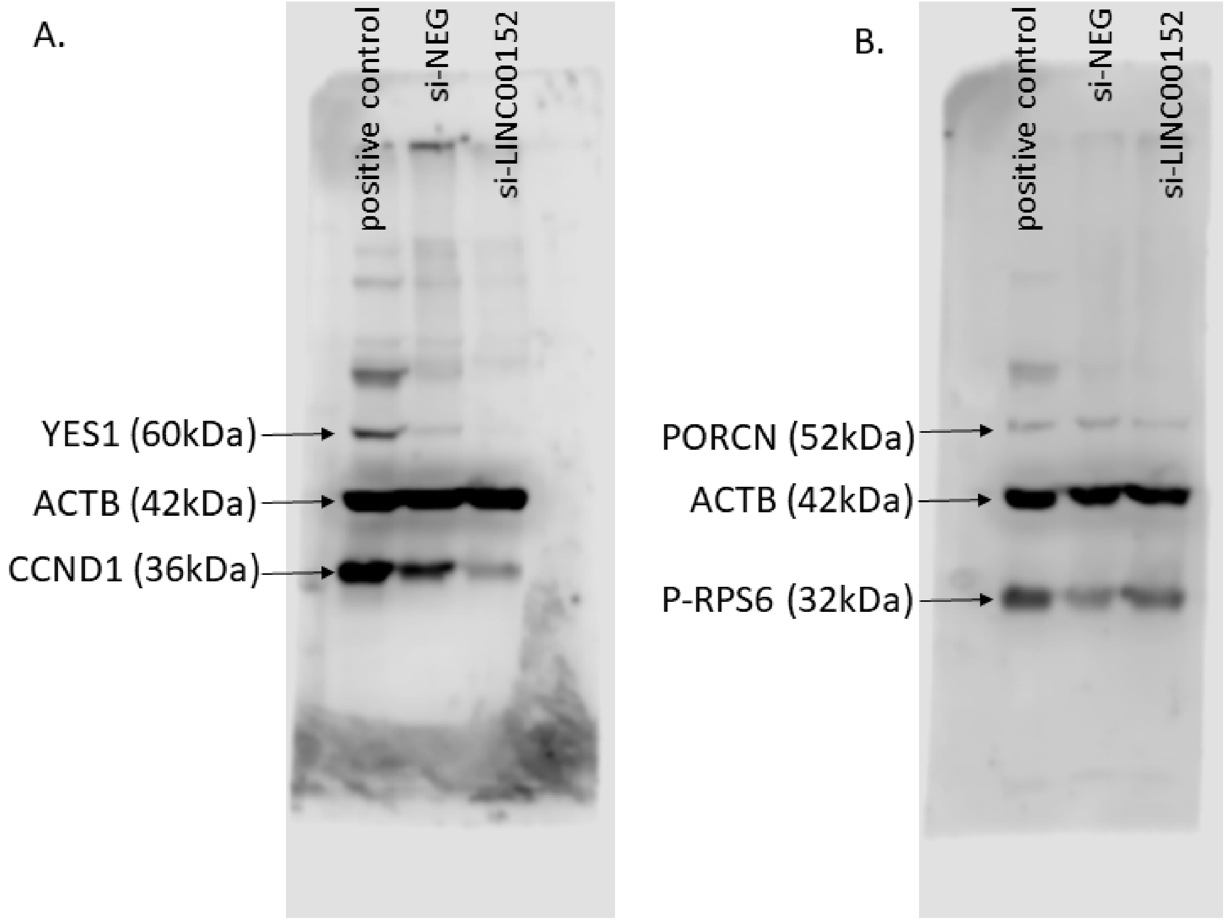

A. Full-length representative western blot images of YES proto-oncogene 1, Src family tyrosine kinase (YES1) and cyclin D1 (CCND1) proteins and **B.** porcupine O-acyltransferase (PORCN) and phosphorylated S6 ribosomal protein (P-RPS6) proteins. As a loading control beta-actin (ACTB) and as positive control protein extract from untreated SW480 colon carcinoma cells were applied.

Supplementary Figure 3. Parallel analyses of CYTOR expression, LINC00152 promoter DNA methylation and CNV of CYTOR coding gene on GDC TCGA COAD (Colon Adenocarcinoma) data set

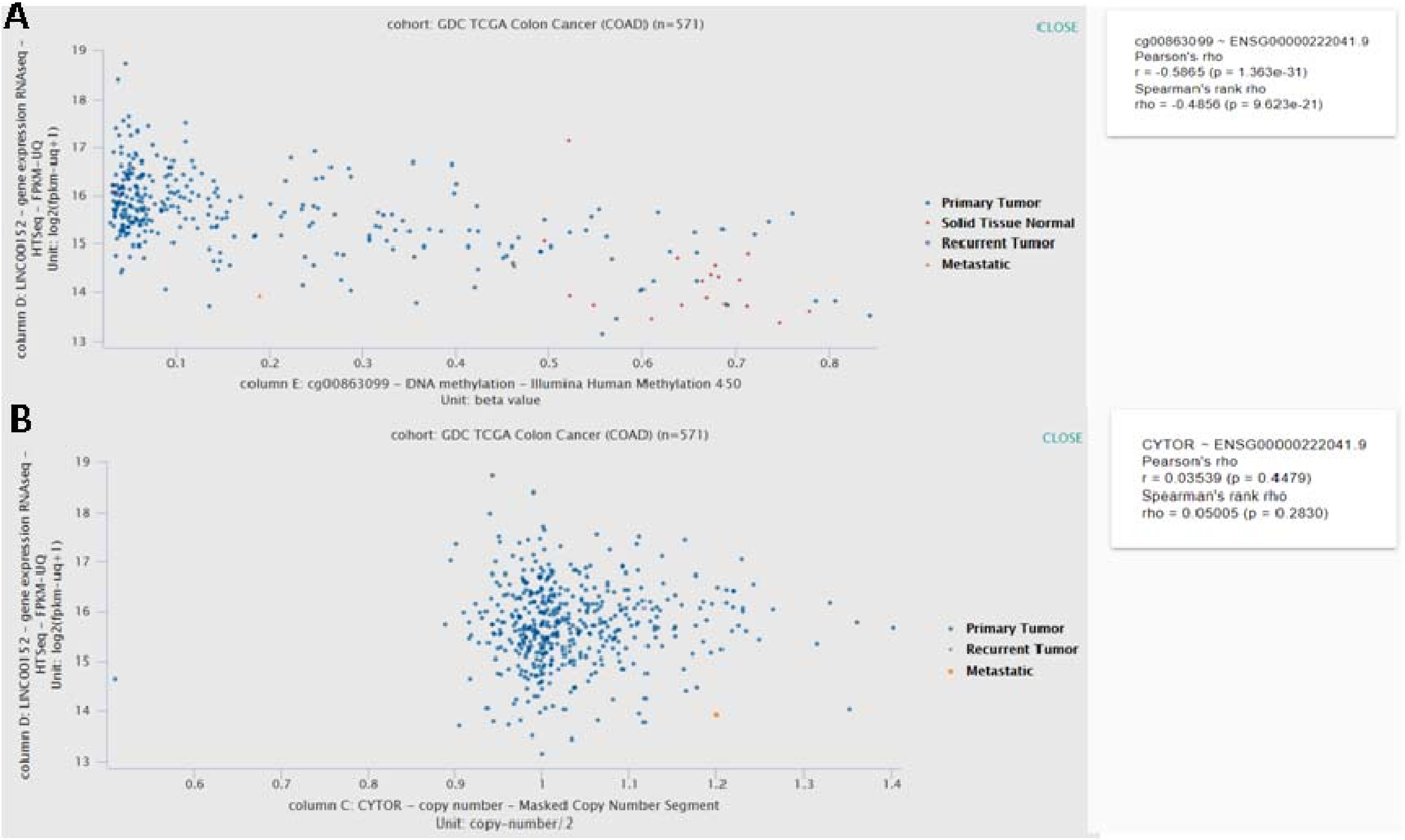

A. Parallel analysis of LINC00152 promoter DNA methylation (BeadChip45K0K, cg00863099 - LINC00152-related CpG site) and CYTOR expression (RNAseq) revealed strong/moderate negative correlation between elevated CYTOR expression and hypomethylation of its promoter (Pearson's rho= -0.5865, p<0.0001). **B.** No significant correlation was found between CYTOR expression levels and CNV of its coding gene (Pearson's rho=0.03539, p=0.4479).

Supplementary Table S1. Clinical data of samples involved in the study and the applied analysis methods

Sample ID	Histology	Age	Gender	TNM	Dukes' Stage	Grade	Dysplasia in adenoma	Localization	HGU133 Plus2.0 array	RT-PCR	HTA2.0 array	ISH - TMA	Methyl capture sequencing	Bisulfite sequencing
									147 samples	90 samples	60 samples	31 patients	30 samples	47 samples
N1024.CEL	normal	47	F					sigmoid	+					
N1081.CEL	normal	57	F					descendent	+					
N1114.CEL	normal	60	F					sigmoid	+					
N1122.CEL	normal	45	M					rectum	+					
N1357.CEL	normal	47	M					ascendent	+					
N1431.CEL	normal	50	M					sigmoid	+					
N1440.CEL	normal	44	F					sigmoid	+					
N1456.CEL	normal	55	M					sigmoid	+					
N2323.CEL	normal	61	F					sigmoid	+					
N2350.CEL	normal	61	M					descendent	+					
N2378.CEL	normal	45	M					sigmoid	+					
N2622.CEL	normal	30	F					sigmoid	+					
N2642 .CEL	normal	57	M					sigmoid	+					
N2647.CEL	normal	36	M					cecum	+	+				
N2664.CEL	normal	49	F					sigmoid	+	+				
N2668.CEL	normal	65	F					rectum	+					
N2675.CEL	normal	46	F					sigmoid	+					
N2689.CEL	normal	54	M					sigmoid	+					
N2691.CEL	normal	77	F					sigmoid	+					
N2701.CEL	normal	27	M					colon	+					
N2770.CEL	normal	82	F					sigmoid	+					
N2771.CEL	normal	62	F					sigmoid	+					
N2781.CEL	normal	74	M					sigmoid	+	+				
N2785.CEL	normal	71	M					sigmoid	+					
N3008.CEL	normal	54	F					sigmoid, rectum	+					
N3020.CEL	normal	62	F					ascendent, sigmoid	+	+				
N3035.CEL	normal	22	M					ascendent, descendent, sigmoid	+	+				
N3038.CEL	normal	73	F					ascendent, sigmoid	+					
N3039.CEL	normal	75	F					sigmoid	+	+				
N3040.CEL	normal	41	M					colon	+					
N3048.CEL	normal	43	F					sigmoid, rectum	+					
N3054.CEL	normal	41	M					sigmoid, rectum	+					
N3057.CEL	normal	30	M					ascendent, rectum	+					

N3062.CEL	normal	66	M					sigmoid	+	+				
N3065.CEL	normal	62	F					sigmoid	+	+				
N3069.CEL	normal	61	F					sigmoid	+					
N3090.CEL	normal	40	F					colon	+	+				
N3103.CEL	normal	41	M					sigmoid, rectum	+					
N3106.CEL	normal	47	F					colon	+					
N3108.CEL	normal	67	M					colon	+					
N3109.CEL	normal	40	F					descendent, rectum	+					
N3112.CEL	normal	44	F					ascendent, transverse, descendent, sigmoid	+					
N3114.CEL	normal	31	F					ascendent, sigmoid	+					
N3115.CEL	normal	82	F					sigmoid, rectum	+					
N3118.CEL	normal	33	F					colon	+					
N3126.CEL	normal	64	F					sigmoid, rectum	+					
N3129.CEL	normal	64	F					cecum, sigmoid	+					
N3140.CEL	normal	45	F					sigmoid	+	+				
N3142.CEL	normal	51	F					sigmoid	+					
AL1138.CEL	tubulovillous adenoma	70	M				low-grade	rectum	+					
AL1154.CEL	tubulovillous adenoma	73	F				low-grade	cecum	+					
AL1419.CEL	villous adenoma	37	F				low-grade	rectum	+					
AL1830.CEL	tubulovillous adenoma	70	M				low-grade	sigmoid	+					
AL2096.CEL	tubulovillous adenoma	82	F				low-grade	descendent	+					
AL2473.CEL	villous adenoma	44	F				low-grade	sigmoid	+					
AL2586.CEL	villous adenoma	84	M				low-grade	ascendent	+					
AL2619.CEL	tubulovillous adenoma	51	F				low-grade	sigmoid	+					
AL2623.CEL	tubulovillous adenoma	61	F				low-grade	cecum	+					
AL2678.CEL	tubular adenoma	71	M				low-grade	descendent	+					
AL2796.CEL	villous adenoma	50	F				low-grade	sigmoid	+					
AL2863.CEL	tubulovillous adenoma	88	F				low-grade	transverse	+	+				
AL2937.CEL	tubular adenoma	57	M				low-grade	rectum	+	+				
AL2957.CEL	tubular adenoma	59	M				low-grade	transverse	+	+				
AL2964.CEL	tubular adenoma	77	M				low-grade	sigmoid	+	+				
AL2982.CEL	tubular adenoma	58	M				low-grade	sigmoid	+	+				
AL2989.CEL	tubular adenoma	73	F				low-grade	ascendent	+	+				

AL3001.CEL	tubulovillous adenoma	75	F				low-grade	sigmoid	+					
AL3026.CEL	tubular adenoma	83	M				low-grade	ascendent	+					
AL3045.CEL	tubulovillous adenoma	65	F				low-grade	sigmoid	+	+				
AL3049.CEL	tubular adenoma	54	M				low-grade	rectosigmoid	+	+				
AL3111.CEL	tubulovillous adenoma	70	M				low-grade	rectum	+					
AL3120.CEL	tubulovillous adenoma	66	M				low-grade	rectum	+	+				
AL980.CEL	tubulovillous adenoma	83	F				low-grade	cecum	+					
AL995.CEL	tubulovillous adenoma	58	M				low-grade	cecum, cecum, rectum	+					
AH1115.CEL	tubulovillous adenoma	75	F				high-grade	cecum	+					
AH1141.CEL	tubulovillous adenoma	63	M				high-grade	rectum	+					
AH1187.CEL	tubulovillous adenoma	80	F				high-grade	cecum	+					
AH1312.CEL	villous adenoma	93	M				high-grade	rectum	+					
AH1700.CEL	tubulovillous adenoma	69	F				high-grade	cecum	+					
AH1748.CEL	villous adenoma	78	F				high-grade	descendent	+					
AH1832.CEL	villous adenoma	56	M				high-grade	sigmoid-descendent	+					
AH2137.CEL	tubulovillous adenoma	82	F				high-grade	descendent	+					
AH2246.CEL	tubulovillous adenoma	73	M				high-grade	cecum	+					
AH2573.CEL	tubulovillous adenoma	59	F				high-grade	rectum	+					
AH2739.CEL	villous adenoma	62	M				high-grade	rectum	+					
AH2803.CEL	tubular adenoma	43	M				high-grade	sigmoid	+					
AH2810.CEL	tubulovillous adenoma	61	M				high-grade	rectum	+					
AH2821.CEL	tubular adenoma	79	F				high-grade	sigmoid	+	+				
AH2916.CEL	villous adenoma	67	M				high-grade	rectum	+					
AH2945.CEL	tubular adenoma	87	F				high-grade	rectum	+					
AH2952.CEL	tubular adenoma	62	M				high-grade	ascendent	+					
AH3051.CEL	tubular adenoma	75	F				high-grade	sigmoid	+					
AH3059.CEL	tubulovillous adenoma	69	M				high-grade	rectum	+					
AH3139.CEL	tubulovillous adenoma	69	M				high-grade	cecum	+					

AH3147.CEL	tubulovillous adenoma	49	F				high-grade	rectum	+					
AH3151.CEL	villous adenoma	78	F				high-grade	transverse	+					
AH956.CEL	villous adenoma	84	F				high-grade	hepatic flexure	+					
AH983.CEL	tubulovillous adenoma	64	F				high-grade	rectum	+					
B1158.CEL	adenocarcinoma	56	M	T3N0M0	B2	G2		rectum	+					
B1293.CEL	adenocarcinoma	88	F	T3N0M0	B2	G3		ascendent	+					
B1486.CEL	adenocarcinoma	51	F	T2N0M0	B1	G1		hepatic flexure	+					
B1708.CEL	adenocarcinoma	72	F	T3N0M0	B2	G2		sigmoid	+					
B1739.CEL	adenocarcinoma	76	F	T4N0M0	B3	G1		descendent	+					
B1761.CEL	adenocarcinoma	58	M	T2N0M0	B1	G2		rectum	+					
B1883.CEL	adenocarcinoma	56	M	T3N0M0	B2	G2		cecum	+					
B2237.CEL	adenocarcinoma	64	M	unknown	B	unknown		rectum	+					
B2382.CEL	adenocarcinoma	68	M	unknown	B	G1		descendent	+					
B2486.CEL	adenocarcinoma	75	F	T2N0M0	B2	G1		sigmoid	+					
B2681.CEL	adenocarcinoma	67	M	T1N0M0	A	G2		rectum	+	+				
B2682.CEL	adenocarcinoma	80	F	T3N0M0	B2	G2		ascendent	+					
B2707.CEL	adenocarcinoma	63	F	T3N0M0	B2	G2		rectum	+					
B2724.CEL	adenocarcinoma	76	F	T3N0M0	B2	G2		cecum	+	+				
B2849.CEL	adenocarcinoma	80	F	T3N0M0	B2	G2		ascendent	+	+				
B2865.CEL	adenocarcinoma	85	M	T2N0M0	B1	G2		cecum	+					
B2900.CEL	adenocarcinoma	79	M	T3N0M0	B2	G2		cecum	+					
B2925.CEL	adenocarcinoma	76	F	T3N0M0	B2	G2		transverse	+					
B2927.CEL	adenocarcinoma	65	F	T2N0M0	B1	G1		rectum	+	+				
B2940.CEL	adenocarcinoma	65	F	T3N0M0	B2	G1		rectum	+	+				
B2956.CEL	adenocarcinoma	67	F	T3N0M0	B2	G2		sigmoid	+					
B2986.CEL	adenocarcinoma	67	F	T2N0M0	B1	G1		ascendent	+					
B2991.CEL	adenocarcinoma	81	F	T3N0M0	B2	G2		ascendent	+					
B3068.CEL	adenocarcinoma	73	M	T3N0M0	B2	G1		lienic flexure	+					
D1146.CEL	adenocarcinoma	46	M	T2N1M0	C1	G2		descendent	+					
D1316.CEL	adenocarcinoma	85	M	T3N1M1	D	G2		lienic flexure	+					
D1377.CEL	adenocarcinoma	69	F	T4N1M0	C3	G1-2		sigmoid	+					
D1479.CEL	adenocarcinoma	77	F	T4N0M1	D	G1		sigmoid	+					
D1494.CEL	adenocarcinoma	73	M	T3N1M1	D	G1		rectum	+					
D1499.CEL	adenocarcinoma	65	M	T3N0M1	D	G1		rectum	+					
D1556.CEL	adenocarcinoma	66	F	T3N1M1	D	G2		cecum	+					
D1651.CEL	adenocarcinoma	59	F	T2N0M1	D	G1		rectum	+					
D2089.CEL	adenocarcinoma	60	M	T3N2M1	D	G1		sigmoid	+					
D2171.CEL	adenocarcinoma	78	M	T3N2M1	D	G3		cecum	+					
D2262.CEL	adenocarcinoma	54	F	T2N1M0	C1	G1		rectum	+					
D2325.CEL	adenocarcinoma	66	M	T2N0-1?	C or B	unknown		rectum	+					

D2651.CEL	adenocarcinoma	55	F	T3N2M0	C3	G2		rectum-sigmoid	+					
D2660.CEL	adenocarcinoma	73	F	T3N2M1	D	G2		rectum	+	+				
D2662.CEL	adenocarcinoma	78	M	T3N2M1	D	G2		ascendent	+					
D2684.CEL	adenocarcinoma	74	F	T3N1M0	C	G2		cecum	+					
D2730.CEL	adenocarcinoma	65	M	T2N1M0	C1	G2		rectum	+					
D2751.CEL	adenocarcinoma	85	F	T3N1M0	C	G2		cecum	+	+				
D2765.CEL	adenocarcinoma	48	M	T4N2M0	C3	G2		rectum	+					
D2842.CEL	adenocarcinoma	66	F	T3N1M0	C	G2		rectum	+	+				
D2872.CEL	adenocarcinoma	61	F	T3N1M0	C2	G2		rectum	+					
D2876.CEL	adenocarcinoma	70	M	T3N1M0	C2	G3		rectum	+					
D2884.CEL	adenocarcinoma	75	F	T3N1M1	D	G2		rectum	+	+				
D2903.CEL	adenocarcinoma	60	M	T3N1M1	D	G2		rectum	+					
D2936.CEL	adenocarcinoma	40	M	T2N3M1	D	G2		rectum	+					
2635	adenocarcinoma	62	F	unknown	unknown	unknown		hepatic flexure		+				
4955	normal	75	F					colon		+	+	+		
4962	normal	63	F					colon		+	+			
4879	normal	25	F					colon		+	+			
4880	normal	60	F					colon		+	+	+		
4981	normal	56	F					colon		+	+	+		
4123	normal	65	F					colon		+	+			
4885	normal	46	M					colon		+	+			
5212	normal	59	F					colon		+	+			+
5225	normal	68	F					colon		+	+			+
5226	normal	24	F					colon		+	+	+		+
5219	normal	76	M					colon		+	+			
5231	normal	71	F					colon		+	+			+
5237	normal	69	F					colon		+	+			+
5238	normal	57	F					colon		+	+			+
5215	normal	54	M					colon		+	+			+
4439	normal	61	F					colon		+	+			
4215	normal	31	M					colon		+	+			+
4115	normal	39	F					colon		+	+			+
5211	normal	31	F					colon		+	+			+
5135	normal	44	F					colon		+	+			+
4538	tubular adenoma	65	M				low-grade	rectum		+	+			
5141	tubular adenoma	63	M				low-grade	ascendent, transverse, rectum		+	+			+
5123	tubular adenoma	64	M				low-grade	transverse, sigmoid, rectum		+	+			+
4759	tubular adenoma	60	M				low-grade	sigmoid		+	+			+
4792	tubular adenoma	88	M				low-grade	ascendent, transverse, sigmoid		+	+			+

5007	tubular adenoma	68	F				low-grade	rectum		+	+	+		+
5081	tubular adenoma	76	M				low-grade	cecum, sigmoid		+	+	+		+
5049	tubular adenoma	77	F				low-grade	rectum-sigmoid		+	+			+
5048	tubular adenoma	60	F				low-grade	sigmoid		+	+	+		+
5025	tubular adenoma	63	F				low-grade	sigmoid		+	+			+
4472	tubulovillous adenoma	42	F				high-grade	sigmoid		+	+			
R9	tubulovillous adenoma	69	M				low-grade	ascendent		+	+			
4944	tubulovillous adenoma	62	M				low-grade	sigmoid, rectum		+	+			
4931	tubulovillous adenoma	61	M				high-grade	sigmoid, rectum		+	+			
4526	tubular adenoma	62	M				low-grade	rectum, descendent		+	+	+		
4744	tubulovillous adenoma	60	M				low.grade	sigmoid		+	+			
R107	tubulovillous adenoma	87	M				low-grade	sigmoid, rectum		+	+			+
5147	tubulovillous adenoma	63	F				low-grade	sigmoid		+	+	+		+
R119	tubulovillous adenoma	63	F				low-grade	sigmoid		+	+	+		+
4720	tubulovillous adenoma	78	M				low-grade	ascendent, sigmoid, rectum		+	+	+		+
4927	adenocarcinoma	62	M	T3N1M0	C2	G2		sigmoid		+	+	+		
4934	adenocarcinoma	74	M	T3N0M0	B2	G2		rectum		+	+	+		
4989	adenocarcinoma	60	M	unknown	C	unknown		cecum		+	+			
4988	adenocarcinoma	61	F	T3N0M0	B2	unknown		rectum		+	+			
R33	adenocarcinoma	73	F	unknown	D	unknown		rectum		+	+	+		
4728	adenocarcinoma	61	M	unknown	C?	unknown		rectum		+	+			
R42	adenocarcinoma	46	M	T3N0M0	B2	G2		rectum		+	+			
4905	adenocarcinoma	60	M	unknown	D	unknown		rectum		+	+			
R45	adenocarcinoma	74	M	unknown	D	unknown		rectum		+	+			
5020	adenocarcinoma	71	F	T3N0M0	B2	G1		descendent		+	+			
R41	adenocarcinoma	76	F	unknown	C	unknown		hepatic flexure		+	+	+		
5108	adenocarcinoma	76	F	T2N0M0	B1	G2		hepatic flexure		+	+	+		+
5128	adenocarcinoma	73	F	T3N2M1	D	G2		sigmoid		+	+	+		+
5194	adenocarcinoma	60	M	inoperab	D	unknown		hepatic flexure		+	+			+
5101	adenocarcinoma	67	F	unknown	unknown	unknown		sigmoid		+	+	+		+
5203	adenocarcinoma	87	F	T3N0M0	B2	G2		sigmoid		+	+	+		+
5201	adenocarcinoma	68	M	T3N0M0	B2	G1		sigmoid, rectum		+	+			+
5181	adenocarcinoma	85	F	T2N0Mx	B1	G2		cecum		+	+	+		+
5179	adenocarcinoma	65	M	T2N0M0	B1	G1		sigmoid		+	+			+

5076	adenocarcinoma	67	M	T3N1M0	C	G1 and G3		transverse		+	+	+		
MCS_20090930_0001_01_100	normal adjacent tissue	74	M					rectum					+	
MCS_20110000_0019_01_100	normal adjacent tissue	76	F					rectum					+	
MCS_20061114 _0020_01_100	normal adjacent tissue	72	M					cecum					+	
MCS_20061113 _0021_01_100	normal adjacent tissue	68	M					sigmoid					+	
MCS_20110000_0022_01_100	normal adjacent tissue	58	M					sigmoid					+	
MCS_20110000_0023_01_100	normal adjacent tissue	68	F					sigmoid and rectum					+	
MCS_20100222_0004_01_210	adenoma	78	M				low-grade	rectum					+	
MCS_20100427_0005_01_210	adenoma	65	M				low-grade	rectum					+	
MCS_20100609_0007_01_210	adenoma	66	M				low-grade	rectum					+	
MCS_20110310_0012_01_210	adenoma	83	M				low-grade	sigmoid					+	
MCS_20110914_0013_01_210	adenoma	55	M				low-grade	descendent, sigmoid					+	
MCS_20120109_0014_01_211	adenoma	81	M				low-grade	sigmoid					+	
MCS_20120308_0015_01_211	adenoma	48	M				low-grade	transverse					+	
MCS_20120320_0016_01_211	adenoma	58	M				low-grade	sigmoid					+	

MCS_20120801_0017_01_211	adenoma	56	F				low-grade	ascendent, transverse					+	
MCS_20091104_0002_01_220	adenoma	60	M				high-grade	rectum-sigmoid					+	
MCS_20100610_0006_01_220	adenoma	68	M				high-grade	descendent					+	
MCS_20100810_0008_01_220	adenoma	73	M				high-grade	sigmoid					+	
MCS_20100923_0009_01_220	adenoma	74	M				high-grade	rectum					+	
MCS_20110111_0010_01_220	adenoma	68	M				high-grade	descendent					+	
MCS_20110203_0011_01_220	adenoma	76	F				high-grade	descendent					+	
MCS_20110203_0011_02_312	adenocarcinoma	76	F	T2N0M0	B1	G2		rectum					+	
MCS_20110310_0012_02_312	adenocarcinoma	83	M	T2N0M0	B1	G2		cecum-ascendent					+	
MCS_20061114 _0020_02_310	adenocarcinoma	72	M	T3N0M0	B2	unknown		cecum					+	
MCS_20061113 _0021_02_310	adenocarcinoma	68	M	T3N0M0	B2	unknown		sigmoid					+	
MCS_20110000_0023_02_311	adenocarcinoma	68	F	T4N0M0	B3	G1		sigmoid and rectum					+	
MCS_20100201_0003_01_320	adenocarcinoma	68	M	unknown	D	unknown		sigmoid-rectum					+	
MCS_20100610_0006_02_322	adenocarcinoma	68	M	T3N2Mx	D	G2		descendent					+	
MCS_201101	adenocarcinoma	68	M	T2N2M0	C2	G2		rectum					+	

11_0010_02_ 322														
MCS_201109 14_0013_02_ 322	adenocarcinoma	55	M	T3N1M0	C2	G2		rectum					+	+
5259	normal	31	F					colon						+
5253	normal	47	M					colon						+
5100	tubular adenoma	73	M				low-grade	cecum, ascendent, descendent						+
5267	tubular, tubulovillous adenoma	63	M				low-grade	rectum						+
4813	tubular adenoma	72	F				low-grade	rectum						+
4883	tubular adenoma	45	F				high-grade	transverse						+
5032	tubular adenoma	65	F				low-grade	ascendent, transverse, rectum						+
5060	tubular adenoma	55	F				low-grade	ascendent						+
5094	tubular adenoma	62	F				high-grade	sigmoid						+
5096	tubulovillous adenoma	83	M				high-grade	ascendent						+
5220	tubulovillous adenoma	68	M				low-grade	rectum, sigmoid						+
5227	adenocarcinoma	72	M	unknown	C	unknown		sigmoid						+
5241	adenocarcinoma	69	M	T3N1M0	C2	G2		rectum						+
5260	adenocarcinoma	73	M	T3N1M0	C1	G2		sigmoid						+
4881	tubulovillous adenoma	74	F				low-grade	cecum, ascendent, sigmoid				+		
4942	tubular adenoma	69	M				low-grade	sigmoid				+		
4967	tubular adenoma	59	F				low-grade	sigmoid				+		
R25	tubular adenoma	63	F				low-grade	cecum, ascendent, sigmoid				+		
4831	tubulovillous adenoma	82	F				low-grade	rectum				+		
4788	tubulovillous adenoma	57	F				low-grade	cecum, ascendent, hepatic flexure				+		
4803	adenocarcinoma	55	M	T3N1M0	C	unknown		rectum				+		
R22	adenocarcinoma	53	M	unknown	D	unknown		transverse				+		
R32	adenocarcinoma	75	F	T3N0M0	B2	unknown		transverse				+		
R44	adenocarcinoma	87	M	unknown	unknown	unknown		rectum				+		

Supplementary Table S2.

S2A. Integrated pathway analysis results of differentially expressed genes (247) in LINC00152-silenced SW480 cells compared to negative control cells

Signalling pathway	Number of genes	Gene symbols
PI3K/AKT	14	FN1*, HSP90B1, ITGA1*, ITGA6*, KPNA1, LAMC1, MET, PRKCA, SOS2, SYK, TBC1D4, THBS1, TP53, YES1*
RAS	10	ARF6, ETS1, MET, PLA2G16, PRKCA, PSMA3, RAB5C, RAB8B, RRAS, SOS2
WNT	9	ATF3, DKK1*, PORCN*, PRKCA, PSMA3, SNAI2*, TP53, WNT5A, YES1*
TP53	8	ATF3, BNIP3L, CSNK1G3, MET, PERP*, PSMA3, SNAI2*, TP53
TGFB	8	ATF3, ETS1, FN1*, MET, SNAI2*, THBS1, TP53, XIAP
ERBB	7	ABI1, CBL, CRK, PRKAR1A, PRKCA, SOS2, YES1*
NOTCH	5	CBL, HES1*, JAG1, RRAS, TP53
IL	5	CCL20*, IL1RAP, LCN2*, SOS2, SYK
cytokine	4	ANXA1*, HSP90B1, NUP37, SAMHD1
TLR	4	CTSS, CXCL8*, MIR3652, SAA1*
Hedgehog	4	CSNK1G3, DERL2*, PSMA3, STIL
MAPK	3	CRK, IL1RAP, RRAS
PDGFR	3	PRKAR1A, PSMA3, YES1*
TCR	2	AHSA1, PSMA3
TNF, TNFR	2	CCL20*, MAP4K5
GPCR	2	PRKAR1A, SAA1*
NOD, NLR	2	XIAP, TNXIP*
ER	2	HSP90B1, MMP9
VEGF	2	PSMA3, YES1*
insulin	2	MAP4K5, TBC1D4
nuclear receptor	2	MED1, NR2F1*
IGF1	1	CRK
MET	1	LAMC1
Rho GTPase	1	RCC2
FGF	1	MMP9
NGF	1	PRKAR1A
CaM	1	PRKAR1A
IFN	1	SAMHD1
NFKB	1	SWAP70
BCR	1	SYK
FAS	1	SYK
Hippo	1	TJP2

Gene symbols marked with * are members of shortened (74 DEGs) list, as well.

S2B. Differentially expressed genes (247) in LINC00152-silenced SW480 cells compared to negative control cells analysed by DAVID 6.8 functional annotation tool (KEGG pathways)

Category	Term	Count	%	p-value	Genes	List Total	Pop Hits	Pop Total	Fold Enrichment	Bonferroni	Benjamini	FDR
KEGG_PATHWAY	hsa05200:Pathways in cancer	14	6,6351	0,001298	WNT5A, PRKCA, XIAP, MMP9, MET, CBL, TP53, CXCL8*, HSP90B1, ITGA6*, SOS2, LAMC1, CRK, FN1*	89	393	6910	2,765817537	0,2125616	0,0765667	1,59278
KEGG_PATHWAY	hsa04151:PI3K-Akt signalling pathway	11	5,2133	0,011871	PRKCA, HSP90B1, ITGA6*, SOS2, MET, TP53, ITGA1*, LAMC1, THBS1, FN1*, SYK	89	345	6910	2,475492591	0,88890151	0,4226661	13,7255
KEGG_PATHWAY	hsa05205:Proteoglycans in cancer	10	4,7393	9,41E-04	PRKCA, WNT5A, MMP9, CBL, SOS2, MET, TP53, RRAS, THBS1, FN1*	89	200	6910	3,882022472	0,15912304	0,159123	1,15769
KEGG_PATHWAY	hsa04510:Focal adhesion	10	4,7393	0,001161	PRKCA, ITGA6*, XIAP, SOS2, MET, ITGA1*, LAMC1, THBS1, CRK, FN1*	89	206	6910	3,768953856	0,19238632	0,1013267	1,42537
KEGG_PATHWAY	hsa04014:Ras signalling pathway	8	3,7915	0,024621	PRKCA, PLA2G16, ETS1, RAB5C, SOS2, MET, RRAS, ARF6	89	226	6910	2,748334493	0,98981673	0,3993048	26,5227
KEGG_PATHWAY	hsa05206:MicroRNAs in cancer	8	3,7915	0,07026	PRKCA, MMP9, SOS2, MET, TP53, THBS1, HMGA2, CRK	89	285	6910	2,179381037	0,99999849	0,6161311	59,3682
KEGG_PATHWAY	hsa04142:Lysosome	6	2,8436	0,018723	SLC17A5, LAPTM5*, NPC2, LGMN, CTSS, CD164	89	121	6910	3,849939642	0,96912332	0,439892	20,8373

KEGG_PATHWAY	hsa04530:Tight junction	6	2,8436	0,030079	PRKCA, CLDN1, CLDN22, RRAS, YES1*, TJP2	89	137	6910	3,400311654	0,99637348	0,4299027	31,4471
KEGG_PATHWAY	hsa04145:Phagosome	6	2,8436	0,045053	STX7, OLR1*, C3, RAB5C, CTSS, THBS1	89	153	6910	3,044723507	0,99979291	0,5375073	43,4428
KEGG_PATHWAY	hsa05222:Small cell lung cancer	5	2,3697	0,022761	ITGA6*, XIAP, TP53, LAMC1, FN1*	89	85	6910	4,567085261	0,98554036	0,4540343	24,7713
KEGG_PATHWAY	hsa04512:ECM-receptor interaction	5	2,3697	0,02455	ITGA6*, ITGA1*, LAMC1, THBS1, FN1*	89	87	6910	4,462094795	0,98967924	0,435435	26,4565
KEGG_PATHWAY	hsa05146:Amoebiasis	5	2,3697	0,045768	PRKCA, RAB5C, CXCL8*, LAMC1, FN1*	89	106	6910	3,662285351	0,99981956	0,5124413	43,9639
KEGG_PATHWAY	hsa05160:Hepatitis C	5	2,3697	0,089102	SOS2, TP53, CLDN1, CLDN22, CXCL8*	89	133	6910	2,918813889	0,99999997	0,6580966	68,4544
KEGG_PATHWAY	hsa04310:Wnt signalling pathway	5	2,3697	0,098743	PRKCA, WNT5A, DKK1*, TP53, PORCN*	89	138	6910	2,813059762	1	0,654495	72,3427
KEGG_PATHWAY	hsa05219:Bladder cancer	4	1,8957	0,015013	MMP9, TP53, CXCL8*, THBS1	89	41	6910	7,574677994	0,93817218	0,4268916	17,0567
KEGG_PATHWAY	hsa05220:Chronic myeloid leukemia	4	1,8957	0,063543	CBL, SOS2, TP53, CRK	89	72	6910	4,313358302	0,99999433	0,6051421	55,5869
KEGG_PATHWAY	hsa05100:Bacterial invasion of epithelial cells	4	1,8957	0,076841	CBL, MET, CRK, FN1*	89	78	6910	3,98156151	0,99999959	0,6249788	62,7847
KEGG_PATHWAY	hsa04666:Fc gamma R-mediated phagocytosis	4	1,8957	0,091259	PRKCA, ARF6, CRK, SYK	89	84	6910	3,697164259	0,99999998	0,6450423	69,3656
KEGG_PATHWAY	hsa04012:ErbB signalling pathway	4	1,8957	0,098862	PRKCA, CBL, SOS2, CRK	89	87	6910	3,569675836	1	0,6350858	72,3878

Gene symbols marked with * are members of shortened (74 DEGs) list, as well.

S2C. Differentially expressed genes (247) in LINC00152-silenced SW480 cells compared to negative control cells analysed by TAC4.0 software (WikiPathways)

Pathway	#Total	#Up	Up List	#Down	Down List	Significance	p-value
miR-targeted genes in muscle cell - TarBase	14	7	THBS1; CLDN1; MET; ARL2; LAMC1; PHLDB2; WNT5A	7	PODXL; RCN2*; UBE2J1; TMEM43; SH3BGRL3*; STX7; CD164	5,1	0,000008
miR-targeted genes in epithelium - TarBase	12	5	CLDN1; MET; LAMC1; PHLDB2; WNT5A	7	PODXL; RAB5C; RCN2*; UBE2J1; TMEM43; STX7; CD164	4,53	0,000029
miR-targeted genes in lymphocytes - TarBase	12	5	MET; WNT5A; ARL2; LAMC1; CLDN1	7	TMEM43; RCN2*; SH3BGRL3*; RAB5C; CD164; STX7; UBE2J1	3,08	0,000824
PI3K-Akt Signalling Pathway	11	7	FN1*; ITGA1*; LAMC1; THBS1; MET; HSP90B1; SYK	4	ITGA6*; SOS2; TP53; PRKCA	4,01	0,000098
Epithelial to mesenchymal transition in colorectal cancer	10	5	JAG1; EIF5A2; CLDN1; FN1*; WNT5A	5	TP53; SOS2; SNAI2*; MMP9; CLDN22	6,17	0,000001
VEGFA-VEGFR2 Signalling Pathway	10	4	ANXA1*; JAG1; DKK1*; CXCL8*	6	CRK; CBL; PRKCA; TXNIP*; ARF6; ETS1	4,62	0,000024
Focal Adhesion	9	5	THBS1; MET; LAMC1; FN1*; ITGA1*	4	ITGA6*; CRK; XIAP; PRKCA	4,49	0,000032
Insulin Signalling	8	1	TBC1D4	7	KIF5B*; ARF6; CRK; CBL; SOS2; PRKCA; MAP4K5	4,3	0,00005
Ras Signalling	8	2	RRAS; MET	6	PLA2G16; SOS2; ETS1; PRKCA; ARF6; RAB5C	3,92	0,000121
miR-targeted genes in squamous cell - TarBase	7	3	THBS1; MET; LAMC1	4	PODXL; UBE2J1; TMEM43; SH3BGRL3*	3,48	0,000335
Focal Adhesion-PI3K-Akt-mTOR-signalling pathway	7	6	FN1*; PELO*; LAMC1; THBS1; MET; HSP90B1	1	ITGA6*	1,96	0,010995
Nuclear Receptors Meta-Pathway	7	1	CCL20*	6	ANGPTL4; SNAI2*; MYOF; ALOX5AP*; HES1*; SCP2	1,82	0,015169
TGF-beta Signalling Pathway	6	3	MET; THBS1; FN1*	3	TP53; ETS1; ATF3	3,11	0,000769
EGF/EGFR Signalling Pathway	6	0		6	CRK; ABI1; SOS2; PRKCA; ARF6; CBL	2,71	0,00196
MET in type 1 papillary renal cell carcinoma	5	1	MET	4	CBL; SOS2; CRK; ETS1	3,96	0,000109
Primary Focal Segmental Glomerulosclerosis FSGS	5	4	JAG1; DKK1*; FAT1*; CLDN1	1	PODXL	3,32	0,000483

Spinal Cord Injury	5	2	CXCL8*; ANXA1*	3	MMP9; PRKCA; TP53	2,6	0,002509
Regulation of Actin Cytoskeleton	5	3	FN1*; RRAS; ITGA1*	2	SOS2; CRK	2,13	0,007377
miR-targeted genes in leukocytes - TarBase	5	1	THBS1	4	RAB5C; TMEM43; SH3BGRL3*; CD164	1,99	0,010322
MAPK Signalling Pathway	5	1	RRAS	4	CRK; SOS2; PRKCA; TP53	1,33	0,047152
miRNA targets in ECM and membrane receptors	4	4	THBS1; LAMC1; FN1*; ITGA1*	0		3,31	0,000494
Kit receptor signalling pathway	4	0		4	CRK; SNAI2*; CBL; PRKCA	2,9	0,001272
Glucocorticoid Receptor Pathway	4	1	CCL20*	3	ALOX5AP*; SNAI2*; ANGPTL4	2,6	0,002521
Signalling Pathways in Glioblastoma	4	1	MET	3	CBL; PRKCA; TP53	2,34	0,004621
Hair Follicle Development: Cytodifferentiation (Part 3 of 3)	4	3	PERP*; DKK1*; WNT5A	1	KLK6*	2,21	0,006131
LncRNA involvement in canonical Wnt signalling and colorectal cancer	4	2	WNT5A; DKK1*	2	ATF3; PORCN*	2,1	0,007938
B Cell Receptor Signalling Pathway	4	1	SYK	3	CRK; ETS1; CBL	2,07	0,008512
DNA Damage Response (only ATM dependent)	4	1	WNT5A	3	TP53; SOS2; SCP2	2,01	0,009739
Wnt Signalling Pathway	4	2	WNT5A; DKK1*	2	PRKCA; PORCN*	1,85	0,014093
Ebola Virus Pathway on Host	4	1	ITGA1*	3	ITGA6*; NPC2; KPNA1	1,71	0,019516
Angiopoietin Like Protein 8 Regulatory Pathway	4	0		4	SOS2; CBL; CRK; MAP4K5	1,66	0,022107
Ectoderm Differentiation	4	1	FOXL1	3	PODXL; STC1*; RAB8B	1,56	0,027279
Breast cancer pathway	4	1	WNT5A	3	TP53; HES1*; SOS2	1,45	0,035212
Canonical and Non-canonical Notch signalling	3	2	JAG1; RRAS	1	HES1*	2,8	0,00157
Inflammatory Response Pathway	3	3	FN1*; LAMC1; THBS1	0		2,64	0,00229
Alpha 6 Beta 4 signalling pathway	3	1	LAMC1	2	ITGA6*; PRKCA	2,64	0,00229
Integrated Lung Cancer Pathway	3	0		3	CRK; CBL; ARF6	2,28	0,005214
ErbB Signalling Pathway	3	0		3	CRK; PRKCA; CBL	1,99	0,010177

Hepatitis C and Hepatocellular Carcinoma	3	0		3	PODXL; MYOF; TP53	1,97	0,01068
Non-small cell lung cancer	3	0		3	PRKCA; TP53; SOS2	1,82	0,015222
Angiogenesis overview	3	0		3	PRKCA; CRK; MMP9	1,8	0,015855
PPAR signalling pathway	3	1	OLR1*	2	ANGPTL4; SCP2	1,77	0,017165
RAC1/PAK1/p38/MMP2 Pathway	3	1	FN1*	2	TP53; CRK	1,72	0,01924
G Protein Signalling Pathways	3	1	RRAS	2	PRKAR1A; PRKCA	1,43	0,037475
Wnt Signalling Pathway and Pluripotency	3	1	WNT5A	2	PRKCA; TP53	1,33	0,047243
Neural Crest Differentiation	3	0		3	SNAI2*; HES1*; ETS1	1,33	0,047243
Integrin-mediated Cell Adhesion	3	1	ITGA1*	2	CRK; ITGA6*	1,33	0,047243
Mammary gland development pathway - Involution (Stage 4 of 4)	2	0		2	TP53; MMP9	2,56	0,002734
H19 action Rb-E2F1 signalling and CDK-Beta-catenin activity	2	1	JAG1	1	MED1	2,21	0,006213
Apoptosis Modulation and Signalling	2	0		2	TP53; XIAP	2,21	0,006213
miR-509-3p alteration of YAP1/ECM axis	2	1	FN1*	1	SNAI2*	2,1	0,007963
Inhibition of exosome biogenesis and secretion by Manumycin A in CRPC cells	2	1	RRAS	1	RAB5C	2,05	0,008912
NOTCH1 regulation of human endothelial cell calcification	2	2	ITGA1*; JAG1	0		2,05	0,008912
TGF-B Signalling in Thyroid Cells for Epithelial-Mesenchymal Transition	2	1	FN1*	1	SNAI2*	2	0,009909
Photodynamic therapy-induced unfolded protein response	2	1	HSP90B1	1	ATF3	1,71	0,019508
Bladder Cancer	2	1	THBS1	1	TP53	1,6	0,025312
miRNA regulation of p53 pathway in prostate cancer	2	1	PERP*	1	TP53	1,57	0,02686

Signalling of Hepatocyte Growth Factor Receptor	2	1	ITGA1*	1	CRK	1,52	0,030067
Photodynamic therapy-induced HIF-1 survival signalling	2	0		2	BNIP3L; TP53	1,45	0,035145
miRs in Muscle Cell Differentiation	2	0		2	PRKAR1A; PRKCA	1,39	0,040529
IL-2 Signalling Pathway	2	1	SYK	1	CBL	1,35	0,044279
Vitamin B12 Disorders	1	0		1	CBL	1,62	0,023746
Ultraconserved region 339 modulation of tumor suppressor microRNAs in cancer	1	0		1	TP53	1,41	0,039264
Sandbox Pathway	1	0		1	TP53	1,41	0,039264
Choline catabolism	1	0		1	SLC44A1	1,33	0,046931

Gene symbols marked with * are members of shortened (74 DEGs) list, as well.

Supplementary Table S3. Relationship between LINC00152 expression and molecular features of colorectal cancer according to the *in silico* analysis of whole genome expression microarray data set GSE39582 [43]

Comparison (sample number)	p-value	median	95% CI for the median	logFC
CIMP+ (92) vs. CIMP- (420)	<0.0001*	CIMP+: 8.95<br>CIMP-: 8.61	8.81-9.04<br>8.51-8.67	0.36
MSI (77) vs. MSS (459)	0.0001*	MSI: 8.81<br>MSS: 8.55	8.70-8.99<br>8.48-8.65	0.38
BRAF_mut (51) vs. BRAF_WT (461)	<0.0001*	BRAF_mut: 9.00<br>BRAF_WT: 8.65	8.81-9.21<br>8.56-8.70	0.56
TP53_mut (190) vs. TP53_WT (161)	0.2430	TP53_mut: 8.65<br>TP53_WT: 8.55	8.53-8.75<br>8.41-8.70	NS
KRAS_mut (217) vs. KRAS_WT (328)	0.1045	KRAS_mut: 8.63<br>KRAS_WT: 8.68	8.46-8.72<br>8.63-8.75	NS

*significant difference (Mann-Whitney test), NS= not significant; Sample numbers are represented in brackets.
